# Global hydro-environmental lake characteristics at high spatial resolution

**DOI:** 10.1038/s41597-022-01425-z

**Published:** 2022-06-23

**Authors:** Bernhard Lehner, Mathis L. Messager, Maartje C. Korver, Simon Linke

**Affiliations:** 1 Department of Geography, McGill University, Montreal, QC H3A 0B9 Canada; 2 RiverLY Research Unit, National Research Institute for Agriculture, Food and Environment (INRAE), 69100 Villeurbanne, France; 3 Australian Rivers Institute, Griffith University, Brisbane, QLD 4111 Australia; 4 CSIRO Land and Water, Brisbane, QLD 4102 Australia

**Keywords:** Limnology, Hydrology, Freshwater ecology, Environmental sciences

## Abstract

Here we introduce the LakeATLAS dataset, which provides a broad range of hydro-environmental characteristics for more than 1.4 million lakes and reservoirs globally with an area of at least 10 ha. LakeATLAS forms part of the larger HydroATLAS data repository and expands the existing datasets of sub-basin and river reach descriptors by adding equivalent information for lakes and reservoirs in a compatible structure. Matching its HydroATLAS counterparts, version 1.0 of LakeATLAS contains data for 56 variables, partitioned into 281 individual attributes and organized in six categories: hydrology; physiography; climate; land cover & use; soils & geology; and anthropogenic influences. LakeATLAS derives these attributes by processing and reformatting original data from well-established global digital maps at 15 arc-second (~500 m) grid cell resolution and assigns the information spatially to each lake by aggregating it within the lake, in a 3-km vicinity buffer around the lake, and/or within the entire upstream drainage area of the lake. The standardized format of LakeATLAS ensures versatile applicability in hydro-ecological assessments from regional to global scales.

## Abstract (summary)

**Table:** 

Measurement(s)	hydro-environmental characteristics • lake • water body • hydrographic feature
Technology Type(s)	digital curation
Sample Characteristic - Environment	freshwater environment • aquatic environment
Sample Characteristic - Location	Earth (planet)

## Background & Summary

Although lakes and reservoirs cover only about 2% of the Earth’s land surface, they represent an essential part of the planet’s bio- and hydrosphere: they store about 90% of the Earth’s unfrozen surface freshwaters^1^, are biodiversity hot-spots^2,3^, and contribute to human societies through diverse services and cultural values, including water and food supply, flood control, recreation, tourism, navigation, and spiritual values^4–7^. In addition, lakes and reservoirs play an important role in controlling global-scale hydrological and biogeochemical processes, in regulating regional climate and weather patterns, and in contributing to the global carbon budget^8–13^.

Despite the importance of lakes and reservoirs, our understanding of their functioning and their sensitivity to anthropogenic alterations at the global scale remains constrained by a historical lack of data regarding their hydro-environmental characteristics. Advances in remote sensing and computing over the past two decades have produced increasingly comprehensive and dynamic global maps of inland surface waters^14–17^. But these datasets, on their own, are limited in their ability to describe surface water characteristics and distinguish among lakes, rivers, floodplains, and other wetlands. To aid in this distinction, a global lake-only database of more than 1.4 million individual lake and reservoir shoreline polygons has been created through extensive manual processing of surface water maps, termed HydroLAKES^18^. However, although HydroLAKES provides basic information on the distribution and geometry of lakes, users in need of additional lake characteristics that shape their ecological and geomorphological functioning — such as climatic conditions, topography, or surrounding land cover — still have to derive or summarize this information from disparate sources.

In an era of global environmental pressures and climate change, researchers, conservation organizations, and decision-makers must adopt a large-scale perspective to understand, manage and conserve lakes and reservoirs^3,19,20^. Yet the inconsistency and inaccessibility of data sources across administrative units and lake basins stand in the way of taking such a perspective by precluding seamless analyses at regional to global scales. Even when data are available and sufficiently consistent, the compilation and harmonization of multiple data sources for a large region typically involves repetitive geospatial procedures, often necessitating the development of new algorithms or software customizations. This is not only a time-consuming process requiring specialized computational skills and resources, but the individual, non-standardized solutions also create results that are difficult to compare. For instance, to spatially correlate data that are processed at different resolutions, extents, or projections, the applied alignment procedures can result in potential artefacts and faulty interpolation. Therefore, baseline datasets providing consistent and homogeneous global coverage are needed to enable and support limnological research and water resource management in a timely manner across the world, particularly in remote areas where little monitoring is available.

Few initiatives to date have compiled and harmonized baseline data on the hydro-environmental attributes of lakes at large scales. As notable regional exceptions, the Lake-Catchment (LakeCat) dataset^21^ provides 135 environmental attributes for all 378,088 lakes in the conterminous U.S. at varying lake catchment scales; LAGOS-NE^22^ provides hydro-environmental attributes and *in situ* measurements of lake water quality for lakes across 17 states in the U.S. Northeast; and the forthcoming LakePulse database will provide attributes for lakes across Canada (https://lakepulse.ca). At the global scale, several databases provide hydro-environmental attributes for a small subset of lakes, or a limited selection of attributes for a larger number of lakes; examples include the International Lake Environment Committee Foundation World Lake Database (https://wldb.ilec.or.jp), LakeNet’s Global Lakes Database (http://www.worldlakes.org/lakes.asp), data compiled by the GloboLakes project (http://www.globolakes.ac.uk), or the Global Lake Database (GLDB)^23^. However, to our knowledge the only existing compilation of attributes for a near-complete global set of lakes (≥10 ha) is the Global Lake area, Climate, and Population dataset (GLCP)^24^. GLCP combines static lake polygons from HydroLAKES with calculated yearly lake surface area and basin-level temperature and precipitation metrics, as well as human population estimates for each of the 1.4 million individual lakes. GLCP is a valuable contribution to the global study of lake and reservoir dynamics as its data on lake surface fluctuations not only address important limitations in the component datasets of HydroLAKES (see *Technical Validation*) but also provide information on seasonal and long-term changes in water quantity which are key to understanding the global interplay between lakes, humans, and hydrology^24^. Nonetheless, GLCP shows some technical constraints associated with the spatial units used for the calculation of basin-level statistics (see *Technical Validation* for more details); and its restricted set of environmental variables limits its applicability when data on a broader range of lake characteristics are required (e.g., land cover, physiography, soil conditions). Also, GLCP derives its environmental variables for an encompassing lake surface and basin area. Yet for some environmental conditions it is important to distinguish whether the information is derived from the entire drainage area of the lake, from within the lake proper (i.e., across the lake surface area), or from within close vicinity around the lake’s shoreline, as each of these zones can provide unique contributions to lake ecosystem functioning and integrity^3,21,25,26^.

**Fig. 1:**
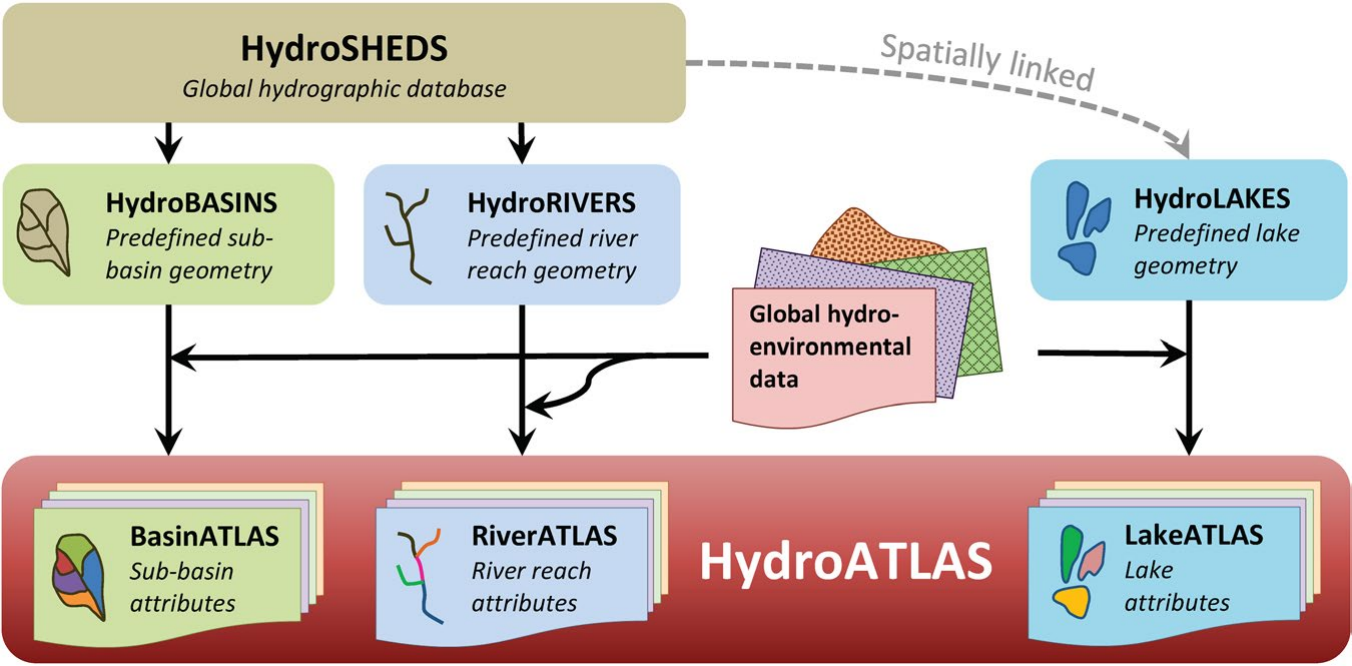
Conceptual design of HydroATLAS and relationship to underpinning HydroSHEDS database. HydroATLAS consists of three companion attribute datasets: BasinATLAS and RiverATLAS (fully described in Linke *et al*.^28^) as well as LakeATLAS (this paper). BasinATLAS provides sub-basin characteristics for hierarchically nested watersheds at twelve spatial scales. RiverATLAS contains the same attributes yet calculated for river and stream reaches rather than sub-basins. The geospatial units for both databases, i.e., sub-basin polygons and river reach line segments, respectively, have been derived from the global hydrographic database HydroSHEDS^39^ at a spatial resolution of 15 arc-seconds (~500 m at the equator). LakeATLAS is added here following the same overall format and structure.

**Fig. 2:**
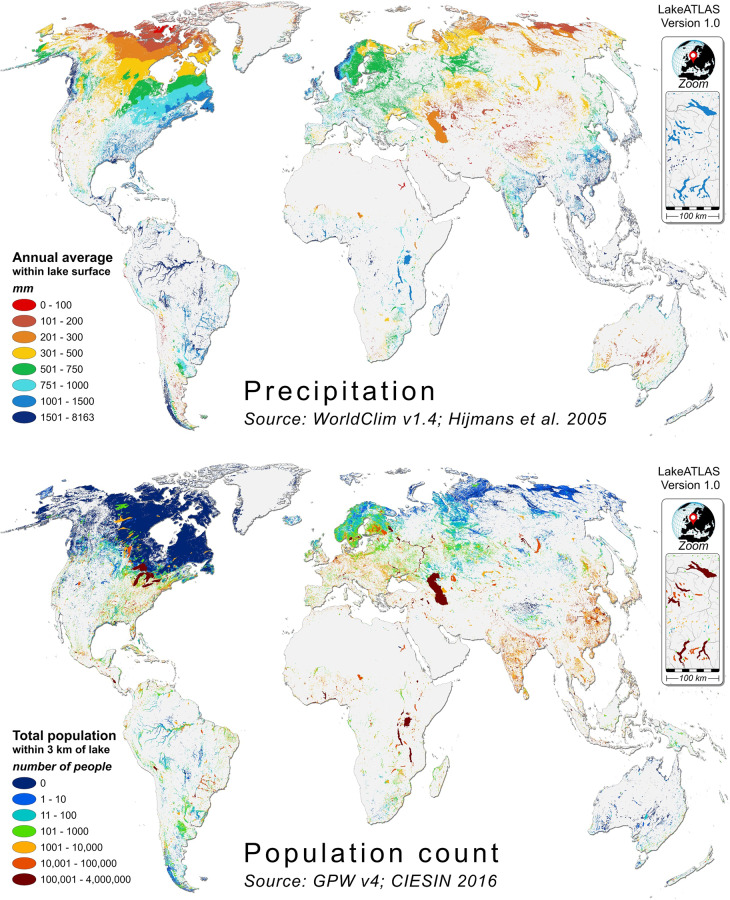
Example attributes of LakeATLAS. Top panel shows average annual precipitation aggregated within the lake surface polygon. Bottom panel shows total population count summed within a 3-km vicinity buffer around each lake. Note that in areas of high lake density, such as in northern Canada, lake surfaces visually appear as contiguous lake regions rather than as distinct lake polygons.

Here we introduce the LakeATLAS dataset^27^, which provides hydro-environmental information for all lakes and reservoirs globally with an area of at least 10 ha that are contained in HydroLAKES. LakeATLAS complements an existing global data repository of hydro-environmental sub-basin and river reach characteristics termed HydroATLAS^28^ (Fig. 1). The goal of the overarching HydroATLAS database is to provide a single, comprehensive, consistently organized and fully global digital data compendium at high spatial resolution, organized in three distinct hydrographic sub-datasets: BasinATLAS (containing ~1.0 million sub-basin polygons), RiverATLAS (containing ~8.5 million river reach lines), and LakeATLAS (containing ~1.4 million lake polygons). The hydro-environmental attributes of all three sub-datasets are compiled from publicly available data sources and are organized in six categories: hydrology; physiography; climate; land cover & use; soils & geology; and anthropogenic influences (Table 1). To match with the remainder of the HydroATLAS database, version 1.0 of LakeATLAS provides the same list of attributes as versions 1.0 of BasinATLAS and RiverATLAS comprising a total of 281 individual attributes that represent 56 different hydro-environmental variables (Table 2 and Fig. 2). The attributes of LakeATLAS are processed for three complementary spatial units: within the lake surface polygon, in a 3-km buffer around the lake, and/or within the entire upstream drainage area of the lake.

**Table 1:** Categories of hydro-environmental characteristics included in LakeATLAS and in the overarching HydroATLAS database (from Linke *et al*.^28^).

Category	Description
Hydrology & hydrography	Hydrological and hydrographic characteristics related to quantity, quality, location, and extent of terrestrial water
	*Examples: natural annual runoff and discharge, lake cover, groundwater table depth*
Physiography	Topographic and geomorphic characteristics related to terrain, relief, or landscape position
	*Examples: elevation, slope, and derivatives*
Climate	Climatic characteristics
	*Examples: mean temperature/precipitation/evaporation, climate moisture index, global aridity*
Land cover & use	Land cover and land use characteristics including biogeographic regions
	*Examples: land cover classes, permafrost extent, freshwater ecoregions*
Soils & geology	Soil and geology related characteristics including substrate types and soil conditions
	*Examples: percent sand/silt/clay in soil, soil water stress, lithography, karst, soil erosion*
Anthropogenic influences	Anthropogenic characteristics and influences including demographic and socioeconomic aspects
	*Examples: population density, human footprint, GDP per capita*

**Table 2:** Hydro-environmental variables and attributes provided in version 1.0 of LakeATLAS (modified from Linke *et al*.^28^).

ID	Category	Variable	Source Data	Source Resolution	Source Year	Reference	Number of Attributes			
							General*		Monthly	
							l	p	v	u
H01	Hydrology	Natural Discharge	WaterGAP v2.2	G: 15 arc-sec	1971–2000^+^	Döll *et al*.^44^		3		
H02	Hydrology	Land Surface Runoff	WaterGAP v2.2	G: 15 arc-sec	1971–2000^+^	Döll *et al*.^44^			1	
H03	Hydrology	Inundation Extent	GIEMS-D15	G: 15 arc-sec	1993–2004	Fluet-Chouinard *et al*.^65^			3	3
H04	Hydrology	Limnicity (percent lake area)	HydroLAKES	V: ~1: 250,000	most recent^$^	Messager *et al*.^18^			1	1
H05	Hydrology	Lake Volume	HydroLAKES	V: ~1: 250,000	most recent^$^	Messager *et al*.^18^				1
H06	Hydrology	Reservoir Volume	GRanD v1.1	V: ~1: 1 million	most recent^$^	Lehner *et al*.^66^				1
H07	Hydrology	Degree of Regulation	HydroSHEDS & GRanD	G: 15 arc-sec	most recent^$^	Lehner *et al*.^66^		1		
H08	Hydrology	River Area	HydroSHEDS & WaterGAP	G: 15 arc-sec	1971–2000^+^	Lehner & Grill^45^			1	1
H09	Hydrology	River Volume	HydroSHEDS & WaterGAP	G: 15 arc-sec	1971–2000^+^	Lehner & Grill^45^			1	1
H10	Hydrology	Groundwater Table Depth	Global Groundwater Map	G: 30 arc-sec	1927–2009^+^	Fan *et al*.^67^			1	
P01	Physiography	Elevation	EarthEnv-DEM90	G: 3 arc-sec	2000–2010	Robinson *et al*.^68^	2		1	1
P02	Physiography	Terrain Slope	EarthEnv-DEM90	G: 3 arc-sec	2000–2010	Robinson *et al*.^68^			1	1
P03	Physiography	Stream Gradient	EarthEnv-DEM90	G: 3 arc-sec	2000–2010	Robinson *et al*.^68^			1	
C01	Climate	Climate Zones	GEnS	G: 30 arc-sec	2000	Metzger *et al*.^69^	1			
C02	Climate	Climate Strata	GEnS	G: 30 arc-sec	2000	Metzger *et al*.^69^	1			
C03	Climate	Air Temperature	WorldClim v1.4	G: 30 arc-sec	1950–2000	Hijmans *et al*.^70^	3|12			1
C04	Climate	Precipitation	WorldClim v1.4	G: 30 arc-sec	1950–2000	Hijmans *et al*.^70^	1|12			1
C05	Climate	Potential Evapotranspiration	Global-PET	G: 30 arc-sec	1950–2000^+^	Zomer *et al*.^71^; Trabucco *et al*.^72^	1|12			1
C06	Climate	Actual Evapotranspiration	Global Soil-Water Balance	G: 30 arc-sec	1950–2000^+^	Trabucco & Zomer^73^			1|12	1
C07	Climate	Global Aridity Index	Global Aridity Index	G: 30 arc-sec	1950–2000^+^	Zomer *et al*.^71^; Trabucco *et al*.^72^	1			1
C08	Climate	Climate Moisture Index	WorldClim & Global-PET	G: 30 arc-sec	1950–2000^+^	Hijmans *et al*.^70^, Zomer *et al*.^71^	1|12			1
C09	Climate	Snow Cover Extent	MODIS/Aqua	G: 15 arc-sec	2002–2015	Hall & Riggs^74^			2|12	1
L01	Land Cover/Use	Land Cover Classes	GLC2000	G: 30 arc-sec	2000	Bartholomé & Belward^75^			1	
L02	Land Cover/Use	Land Cover Extent	GLC2000	G: 30 arc-sec	2000	Bartholomé & Belward^75^			22	22
L03	Land Cover/Use	Potential Natural Vegetation Classes	EarthStat	G: 5 arc-min	1700	Ramankutty & Foley^76^			1	
L04	Land Cover/Use	Potential Natural Vegetation Extent	EarthStat	G: 5 arc-min	1700	Ramankutty & Foley^76^			15	15
L05	Land Cover/Use	Wetland Classes	GLWD	G: 30 arc-sec	historic	Lehner & Döll^77^			1	
L06	Land Cover/Use	Wetland Extent	GLWD	G: 30 arc-sec	historic	Lehner & Döll^77^			11	11
L07	Land Cover/Use	Forest Cover Extent	GLC2000	G: 30 arc-sec	2000	Bartholomé & Belward^75^			1	1
L08	Land Cover/Use	Cropland Extent	EarthStat	G: 5 arc-min	2000	Ramankutty *et al*.^78^			1	1
L09	Land Cover/Use	Pasture Extent	EarthStat	G: 5 arc-min	2000	Ramankutty *et al*.^78^			1	1
L10	Land Cover/Use	Irrigated Area Extent	HID v1.0	G: 5 arc-min	2005	Siebert *et al*.^79^			1	1
L11	Land Cover/Use	Glacier Extent	GLIMS	V: unspecified	1950–2015	GLIMS & NSIDC^80^			1	1
L12	Land Cover/Use	Permafrost Extent	PZI	G: 30 arc-sec	1961–1990^+^	Gruber^81^			1	1
L13	Land Cover/Use	Protected Area Extent	WDPA	V: varying	most recent^$^	UNEP-WCMC & IUCN^82^	1			1
L14	Land Cover/Use	Terrestrial Biomes	TEOW	V: ~1: 1 million	most recent^$^	Dinerstein *et al*.^83^	1			
L15	Land Cover/Use	Terrestrial Ecoregions	TEOW	V: ~1: 1 million	most recent^$^	Dinerstein *et al*.^83^	1			
L16	Land Cover/Use	Freshwater Major Habitat Types	FEOW	V: ~1: 1 million	most recent^$^	Abell *et al*.^84^	1			
L17	Land Cover/Use	Freshwater Ecoregions	FEOW	V: ~1: 1 million	most recent^$^	Abell *et al*.^84^	1			
S01	Soils & Geology	Clay Fraction in Soil	SoilGrids1km	G: 30 arc-sec	most recent^+^	Hengl *et al*.^85^			1	1
S02	Soils & Geology	Silt Fraction in Soil	SoilGrids1km	G: 30 arc-sec	most recent^+^	Hengl *et al*.^85^			1	1
S03	Soils & Geology	Sand Fraction in Soil	SoilGrids1km	G: 30 arc-sec	most recent^+^	Hengl *et al*.^85^			1	1
S04	Soils & Geology	Organic Carbon Content in Soil	SoilGrids1km	G: 30 arc-sec	most recent^+^	Hengl *et al*.^85^			1	1
S05	Soils & Geology	Soil Water Content	Global Soil-Water Balance	G: 30 arc-sec	1950–2000^+^	Trabucco & Zomer^73^			1|12	1
S06	Soils & Geology	Lithological Classes	GLiM	G: 0.5 degrees	1965–2012	Hartmann & Moosdorf^86^			1	
S07	Soils & Geology	Karst Area Extent	Rock Outcrops v3.0	V: unspecified	most recent^$^	Williams & Ford^87^			1	1
S08	Soils & Geology	Soil Erosion	GloSEM v1.2	G: 7.5 arc-sec	2012	Borrelli *et al*.^88^			1	1
A01	Anthropogenic	Population Count	GPW v4	G: 30 arc-sec	2010	CIESIN & SEDAC^61^			1	1
A02	Anthropogenic	Population Density	GPW v4	G: 30 arc-sec	2010	CIESIN & SEDAC^61^			1	1
A03	Anthropogenic	Urban Extent	GHS S-MOD v1.0 (2016)	G: 1 km	2015^+^	Pesaresi & Freire^89^			1	1
A04	Anthropogenic	Nighttime Lights	Nighttime Lights v4	G: 30 arc-sec	2008	Doll^90^			1	1
A05	Anthropogenic	Road Density	GRIP v4	G: 5 arc-min	>1997^$^	Meijer *et al*.^91^			1	1
A06	Anthropogenic	Human Footprint	Human Footprint v2	G: 1 km	1993 & 2009	Venter *et al*.^92^			2	2
A07	Anthropogenic	Global Administrative Areas	GADM v2.0	V: unspecified	2012	University of Berkeley^93^	1			
A08	Anthropogenic	Gross Domestic Product	GDP PPP v2	G: 5 arc-min	2015	Kummu *et al*.^94^			2	1
A09	Anthropogenic	Human Development Index	HDI v2	G: 5 arc-min	2015	Kummu *et al*.^94^			1	
		**Σ = 56 (Variables)**				**Σ = 281 (Asttributes)**	**65**	**4**	**125**	**87**

Abbreviations in column ‘Source Resolution’: G = grid format, V = vector format. Column ‘Number of Attributes’ provides the number of available general attributes (and monthly attributes where present) per spatial aggregation unit: l = within lake, p = at pour point, v = in vicinity, u = upstream. For more information on the definition of variables see the Technical Documentation of LakeATLAS.

*May include different attributes associated with individual classes, or with average, minimum, and/or maximum values.

^$^Data have been compiled from various sources with varying or unknown dates, but are supposed to resemble contemporary/most recent conditions.

^+^Model-based.

Table 3 provides an overview of the main statistical characteristics of each variable as derived from all lakes included in LakeATLAS. We find that lakes occur in all major climate zones, land cover types, biomes, and countries; their distribution spans elevations between −414 and 5,826 m a.s.l. and mean air temperatures between −25.9 and 31.9 °C; in average 29.0% of their upstream area is forested and 12.6% of their upstream area is protected; and we estimate that a total of 965 million people (more than 12% of the world’s population) live within 3 km of a lake.

**Table 3:** Statistical characteristics of selected hydro-environmental variables in LakeATLAS.

ID	Category	Variable	Location	Statistic	Unit	Mean	Median	Min	Max	Mean	Mean
										≥ 100 km^2^	upstream
H01	Hydrology	Natural Discharge	Pour point	Annual mean	m^3^ s^−1^	5.8	0.02	0.0	161,807	340.2	*n.a*.
H02	Hydrology	Land Surface Runoff	3-km vicinity	Annual mean	mm yr^−1^	352.1	256.0	0.0	6,741	335.5	*n.a*.
H03	Hydrology	Inundation Extent	3-km vicinity	Annual max.	%	24.5	2.0	0.0	100.0	43.6	19.4
H04	Hydrology	Limnicity (percent lake area)	3-km vicinity	Spatial extent	%	7.5	5.2	0.0	93.0	13.9	14.9
H05	Hydrology	Lake Volume	Upstream	Sum	10^6^ m^3^	811.9	1.0	0.0	24.2 × 10^6^	220,257	*n.a*.
H06	Hydrology	Reservoir Volume	Upstream	Sum	10^6^ m^3^	28.7	0.0	0.0	310,635	6,536	*n.a*.
H07	Hydrology	Degree of Regulation	Pour point	Value	%	1.2	0.0	0.0	1,000	44.2	*n.a*.
H08	Hydrology	River Area	3-km vicinity	Sum	ha	5.0	0.56	0.0	8,975	266.1	171.6
H09	Hydrology	River Volume	3-km vicinity	Sum	10^3^ m^3^	225.8	0.74	0.0	948,536	9,646	4,514
H10	Hydrology	Groundwater Table Depth	3-km vicinity	Mean	cm	89	57	0	2,967	126	*n.a*.
P01	Physiography	Elevation	Inside lake	Mean	m a.s.l.	281	209	−414	5,826	508	311
P02	Physiography	Terrain Slope	3-km vicinity	Mean	degrees	2.9	2.0	0.0	49.9	3.0	3.1
P03	Physiography	Stream gradient	3-km vicinity	Mean	m km^−1^	6.9	3.0	0.0	632.8	6.0	*n.a*.
C03	Climate	Air Temperature	Inside lake	Annual mean	°C	−3.0	−5.2	−25.9	31.9	6.2	−3.2
C04	Climate	Precipitation	Inside lake	Annual mean	mm yr^−1^	555	434	0	8,163	688	560
C05	Climate	Potential Evapotranspiration	Inside lake	Annual mean	mm yr^−1^	550	438	66	2,282	911	546
C06	Climate	Actual Evapotranspiration	3-km vicinity	Annual mean	mm yr^−1^	377	320	0	1,893	492	377
C07	Climate	Global Aridity Index	Inside lake	Annual mean	—	1.06	0.92	0.0	13.0	0.84	1.08
C08	Climate	Climate Moisture Index	Inside lake	Annual mean	—	−0.05	−0.08	−1.00	0.92	−0.21	−0.04
C09	Climate	Snow Cover Extent	3-km vicinity	Annual mean	%	52.2	59.0	0.0	100.0	30.2	52.5
L07	Land Cover/Use	Forest Cover Extent	3-km vicinity	Spatial extent	%	29.0	0.0	0.0	100.0	26.6	29.0
L08	Land Cover/Use	Cropland Extent	3-km vicinity	Spatial extent	%	4.6	0.0	0.0	100.0	9.7	4.6
L09	Land Cover/Use	Pasture Extent	3-km vicinity	Spatial extent	%	4.5	0.0	0.0	100.0	13.8	4.5
L10	Land Cover/Use	Irrigated Area Extent	3-km vicinity	Spatial extent	%	0.88	0.0	0.0	100.0	1.6	0.8
L11	Land Cover/Use	Glacier Extent	3-km vicinity	Spatial extent	%	0.14	0.0	0.0	100.0	0.08	0.19
L12	Land Cover/Use	Permafrost Extent	3-km vicinity	Spatial extent	%	50.8	56.0	0.0	100.0	21.3	51.3
L13	Land Cover/Use	Protected Area Extent	Inside lake	Spatial extent	%	12.9	0.0	0.0	100.0	17.0	12.6
S01	Soils & Geology	Clay Fraction in Soil	3-km vicinity	Mean	%	12.6	12.0	1.0	45.0	16.0	12.5
S02	Soils & Geology	Silt Fraction in Soil	3-km vicinity	Mean	%	36.5	37.0	2.0	65.0	33.4	36.3
S03	Soils & Geology	Sand Fraction in Soil	3-km vicinity	Mean	%	51.0	50.0	12.0	90.0	50.5	50.9
S04	Soils & Geology	Org. Carbon Content in Soil	3-km vicinity	Mean	tonnes ha^−1^	112.6	109.0	0.0	967.0	72.4	112.3
S05	Soils & Geology	Soil Water Content	3-km vicinity	Annual mean	%	78.4	83.0	0.0	100.0	65.3	78.7
S07	Soils & Geology	Karst Area Extent	3-km vicinity	Spatial extent	%	11.0	0.0	0.0	100.0	10.3	11.1
S08	Soils & Geology	Soil Erosion	3-km vicinity	Mean	kg ha^−1^ yr^−1^	848	105	0.0	146,006	1,861	918
A01	Anthropogenic	Population Count	3-km vicinity	Sum	10^3^ ppl	0.68	0.0	0.0	3,947	36.2	25.6
A02	Anthropogenic	Population Density	3-km vicinity	Mean	ppl km^−2^	27.0	0.0	0.0	61,544	53.9	24.5
A03	Anthropogenic	Urban Extent	3-km vicinity	Spatial extent	%	1.0	0.0	0.0	100.0	2.2	0.83
A04	Anthropogenic	Nighttime Lights	3-km vicinity	Mean	—	2.1	0.0	0.0	63	3.7	1.9
A05	Anthropogenic	Road Density	3-km vicinity	Mean	m km^−2^	95.5	0.0	0.0	330,306	212.3	87.7
A06	Anthropogenic	Human Footprint	3-km vicinity	Year 2009	—	2.4	0.0	0.0	47.9	5.3	2.3
A08	Anthropogenic	Gross Domestic Product	3-km vicinity	Mean	US dollars	52,847	44,791	378	189,535	35,927	*n.a*.
A09	Anthropogenic	Human Development Index	3-km vicinity	Mean	—	0.87	0.90	0.32	1.00	0.81	*n.a*.
**ID**	**Category**	**Variable**	**Location**	**Most frequent majority class**				Least frequent majority class(es)*			
C01	Climate	Climate Zones	Inside lake	Extremely cold and mesic (n = 777,475)				Arctic 1 (n = 0); Arctic 2 (n = 13)			
C02	Climate	Climate Strata	Inside lake	F5 (n = 147,484)				A1 and A2 (n = 0); B3 (n = 2)			
L01	Land Cover/Use	Land Cover Classes	3-km vicinity	Sparse herbaceous/shrub cover (n = 449,656)				Tree cover, regularly flooded, saline (n = 985)			
L03	Land Cover/Use	Potential Nat. Veget. Classes	3-km vicinity	Evergreen/deciduous mixed forest (n = 424,106)				Temperate broadleaf evergreen forest (n = 4,310)			
L05	Land Cover/Use	Wetland Classes	3-km vicinity	Lake (n = 218,019)				Reservoir (n = 2,397)			
L14	Land Cover/Use	Terrestrial Biomes	Inside lake	Boreal forests/taiga (n = 608,381)				Tropical/subtropical coniferous forests (n = 588)			
L15	Land Cover/Use	Terrestrial Ecoregions	Inside lake	Canadian Low Arctic tundra (n = 164,550)				98 terrestrial ecoregions with no lakes			
L16	Land Cover/Use	Freshw. Maj. Habitat Types	Inside lake	Polar freshwaters (n = 773,446)				Oceanic islands (n = 48)			
L17	Land Cover/Use	Freshwater Ecoregions	Inside lake	Eastern Hudson Bay – Ungava (n = 163,795)				34 freshwater ecoregions with no lakes			
S06	Soils & Geology	Lithological Classes	3-km vicinity	Metamorphic rocks (n = 401,414)				Evaporites (n = 1,299)			
A07	Anthropogenic	Global Administrative Areas	Inside lake	Canada (n = 879,900)				24 (small) countries with no lakes			

Statistics in columns ‘Mean’, ‘Median’, ‘Min’, and ‘Max’ were calculated for the full set of 1.4 million lakes contained in LakeATLAS. Column ‘Mean ≥100 km^2^’ refers to the subset of large lakes exceeding 100 km^2^ in surface area (n = 1,708). Column ‘Mean upstream’ was calculated by averaging the ‘upstream’ values for all lakes (i.e., the values derived for the entire drainage area of each lake). Upper panel shows results for numerical data, lower panel shows results for categorical data. Variables not included in this table represent variations of the listed variables, such as individual land cover classes. For more information on the definition of variables see the Technical Documentation of LakeATLAS.

*Excluding ‘no data’ classes.

A unique feature of HydroATLAS and its three companion datasets is the ability to spatially integrate the respective hydrographic features using standard Geographic Information System (GIS) technology. For example, each lake polygon is associated to the river network of RiverATLAS and the sub-basin network of BasinATLAS (via unique IDs), which allows to geolocate each lake within the drainage network. This facilitates topological assessments such as identifying and analyzing the sub-basins or river reaches that are upstream or downstream of a given lake. Using this functionality, additional attributes or advanced statistics can be calculated.

The LakeATLAS dataset is expected to create novel opportunities for a mix of theoretical and applied limnological studies, enabling multi-variable statistical assessments and model-based analyses, and to stimulate large-scale limnological research in otherwise data-poor or remote regions. For example, LakeATLAS can facilitate large-scale assessments of anthropogenic pressures on lakes^29^, support systematic lake classification efforts^30,31^, and reveal biases in monitoring and conservation networks^32,33^, tasks previously unachievable at this scale in the absence of consistent global data. Globally comprehensive data on lake characteristics can also be combined with datasets from *in situ* monitoring networks like the Global Lake Ecological Observatory Network (GLEON)^34^, long-term ecological research networks^35^, other data compilation efforts (e.g., ref. ^36^), or information derived from remote sensing imagery (e.g., refs. ^15,17^) to yield novel insights and refined estimates of the magnitude and distribution of lake processes worldwide. Finally, by connecting individual lakes to the river and stream network and their associated drainage areas, the overarching HydroATLAS database enables advanced mechanistic macroscale analyses of inland waters whereby the fluxes of materials and organisms among the elements of the waterscape can be explicitly modeled^3^.

It should be noted that every effort is made throughout this article to distinguish ‘natural lakes’ from ‘human-made reservoirs’, yet the general term ‘lake’ is used in all instances where this distinction is considered non-critical. Also, the expressions ‘catchment’ and ‘watershed’ are used interchangeably in this article to describe the lake drainage area.

## Methods

### Lake polygons and morphometric attributes

The geospatial foundation of LakeATLAS is formed by the HydroLAKES dataset (https://www.hydrosheds.org/hydrolakes) which provides lake polygons and basic morphometric attributes (e.g., area, depth, volume) for all lakes globally with a surface area of at least 10 ha, comprising a total of 1,427,688 individual lakes^18^. HydroLAKES contains both freshwater and saline lakes, including the Caspian Sea, as well as human-made reservoirs and regulated lakes. HydroLAKES polygons and morphometric attributes were used in their original form for LakeATLAS. This section and the following (*Spatial association of lake polygons with drainage network*) include a brief description of the original production process implemented by Messager *et al*.^18^ — for more details, see the original publication^18^ and the associated Technical Documentation^37^. HydroLAKES was built with the aim to be as comprehensive and consistent as possible at a global scale. It was created by compiling, correcting, and unifying several near-global and regional datasets (see ref. ^37^ for more details) — foremost the Shuttle Radar Topography Mission (SRTM) Water Body Data (SWBD)^14^ for regions from 56°S to 60°N, and CanVec^38^ for lakes in Canada (62% of all lakes in the database). To ensure spatial consistency among the different data sources, map generalization methods were applied to the original polygons and some polygon outlines were smoothed. The resulting map scale is estimated to be between 1:100,000 and 1:250,000 for most lakes globally, with some polygons having a coarser scale of 1:1 million (mostly in Russia north of 60°N).

Each lake polygon in HydroLAKES is associated with a suite of geometric attributes, including lake area, shoreline length, average depth, and volume. Lake area and shoreline length were directly calculated based on the geometry of each lake polygon. Average lake depth and volume were estimated with a geostatistical model based on surrounding land surface topography for all lakes with a surface area under 500 km^2^, and from values reported in the literature for the other 347 lakes over 500 km^2^ in size^18^. Lakes are also distinguished by type (natural lake, artificial reservoir, or natural lake with human-made regulation structure), although ‘natural lake’ was assigned by default unless another type could be confirmed.

### Spatial association of lake polygons with drainage network

HydroLAKES was spatially associated with hydrographic baseline information from the HydroSHEDS raster database^39^ (Fig. 1). This allows for linking each lake to the drainage network by its pour point (the most downstream pixel that drains the lake) and delineating its upstream watershed area. HydroSHEDS consists of a hydrologically conditioned digital elevation model and a corresponding drainage direction map from which auxiliary layers were derived, including flow accumulations, flow distances, river orders, watershed boundaries, and stream networks^39^. HydroSHEDS was initially derived from elevation data of the Shuttle Radar Topography Mission (SRTM)^14,40^ at a pixel resolution of 3 arc-seconds (~90 m at the equator) and was subsequently upscaled to a resolution of 15 arc-seconds (~500 m at the equator). More information on HydroSHEDS is provided at https://www.hydrosheds.org.

HydroLAKES also utilizes river discharge estimates from the global WaterGAP model (version 2.2 as of 2014)^41^, a well-documented and validated integrated water balance model^42–44^. Each 15 arc-second pixel in HydroSHEDS was associated with an estimated long-term (1971–2000) average naturalized discharge value derived from WaterGAP’s 0.5-degree resolution runoff and discharge layers through geospatial downscaling^45^. See the *Technical Validation* section for more details and a quality assessment of the resulting discharge estimates.

To link lake polygons to HydroSHEDS and delineate their upstream drainage area, the most downstream pixel that drains each lake was identified as its pour point^18^. This pour point (or lake outlet) pixel is typically near the lake’s shoreline but can also occur near the center of a lake polygon for terminal lakes in endorheic basins (which have no outlet). To create a single pour point for each lake, the cell accumulation values (i.e., the number of upstream pixels as provided by HydroSHEDS, representing a proxy for watershed area) were analyzed within each lake at a resolution of 15 arc-seconds. Note that in this step, all 15 arc-second pixels that fully or partially (≥1%) overlapped with a lake polygon were considered as candidate pour point pixels. Then, the pixel with the maximum accumulation value per lake was identified as the lake’s pour point. Where multiple pixels existed with equal maximum cell accumulations for the same lake, the pixel with the highest modeled long-term mean annual discharge value (from WaterGAP) was selected. If there were still multiple pixels, a random choice was made among them. This random choice was necessary for only 3.8% of lakes, all small ones (≤6 km^2^ in size) and mostly with low discharge (i.e., 94% of affected lakes presented a mean annual flow at the outlet of less than 0.1 m^3^ s^−1^). This ultimately resulted in one pour point pixel per lake. Finally, the precise coordinates of the pour point location were calculated as the centroid of the intersection between the lake polygon and the pour point pixel, assuring that all pour points are located inside their corresponding lake polygon.

After creating the lake pour points, the upstream watershed area from HydroSHEDS (corrected for latitudinal pixel distortions due to the applied geographic projection) as well as the downscaled discharge estimates were extracted at the location of each lake pour point and assigned to the lake polygon. An estimate of the average residence time for each lake was calculated as the ratio between lake volume and discharge^18^.

### Acquisition and selection of hydro-environmentally relevant attribute data

LakeATLAS relies on the same sources of hydro-environmental data as RiverATLAS and BasinATLAS^28^ (Fig. 1 and Table 2). These input data were acquired either from free and publicly available sources, or from collaborators who provided their data for this project. All data sources were originally assessed regarding their suitability for the entire HydroATLAS project using the following selection criteria:

- completeness of global coverage (allowing only for minor spatial gaps, such as small remote islands, or omission of non-critical areas, such as Greenland or deserts);
- consistency in data quality (i.e., no regional or local biases);
- sufficiency of the native resolution, precision, and accuracy (i.e., resolution of the source data should be in the range of 15 arc-seconds, or finer); and
- permission to use and distribute derivatives under a free license.

If multiple datasets were available for the same attribute, priority was given to the most widely recognized and/or best resolution and/or most recent dataset on a case-by-case basis^28^. It should be noted, however, that the selection of an attribute dataset does not imply any kind of endorsement or warranty of its quality or superiority over other data. In addition, we acknowledge that new and improved sources of data have been published since the creation of RiverATLAS and BasinATLAS (e.g., an updated WordClim climatological database^46^, an updated SoilGrids pedological database^47^, and new/updated global land cover products^48,49^), yet we opted to keep all data sources originally included in HydroATLAS to warrant consistency and the capacity to link and compare attributes across the three datasets. Data sources will be updated simultaneously for the three datasets in future versions of HydroATLAS.

### Preprocessing of attribute data

Before extracting their attribute information into LakeATLAS format, the original attribute datasets were preprocessed into a standardized grid format with the same geometric specifications as the HydroSHEDS 15 arc-second resolution grids. Standardization was applied to datasets in both raster (i.e., representing data as a grid) and vector (i.e., representing data as points, lines, or polygons) formats. The aim of this step was to ensure full spatial congruency between (preprocessed) attribute data and HydroSHEDS, thereby avoiding misalignments in the subsequent conversion processes. All original datasets had already been standardized for the extraction of attributes at the scale of sub-basins (for BasinATLAS) and river reaches (for RiverATLAS), so the same preprocessed grids were used for extracting attributes for LakeATLAS. We provide a brief description of the general workflow below; a full description is available in Linke *et al*.^28^. Additional details for each individual attribute, including on format and resolution, are provided in the Technical Documentation accompanying the LakeATLAS dataset.

The production of standardized attribute grids involved: re-projecting original data sources to a geographic coordinate system with the horizontal datum of the World Geodetic System 1984 (GCS_WGS_1984); converting to grids those datasets that were originally in vector format; aggregating or resampling grids to 15 arc-seconds if needed; cropping or interpolating grids to address mismatches with HydroSHEDS along the coastlines (i.e., layers showing some pixel values in the ocean or lacking pixels on land compared to HydroSHEDS); and filling voids to replace small areas lacking data with spatially interpolated values (though large areas encompassing entire regions or biomes without data were kept unaltered, e.g., if they covered all of Greenland or large deserts regions).

Through these preprocessing steps, all attribute datasets were standardized to the following target grid specifications: a global extent of 180°W to 180°E in longitude and 84°N to 56°S in latitude; a cell size of 15 arc-seconds; a global projection defined by GCS_WGS_1984; and a land-ocean distribution of pixels following the land mask of HydroSHEDS.

### Calculation of statistics within lakes and in their vicinity (‘local’ statistics)

After preprocessing the data sources of hydro-environmental attributes into standardized grids, their values were assigned to individual lakes using one, or more, of three different ‘local’ extraction options (depending on the nature of the attribute): values were extracted either at the lake’s representative pour point; or as a spatial aggregation across the entire surface area of the lake (i.e., the area within its shoreline boundary); or as a spatial aggregation across the area in the lake’s close vicinity (i.e., in a 3-km buffer around the lake). The three different extraction zones were implemented as described below, and the resulting spatial statistics were joined as new attribute columns to the vector layer of HydroLAKES. Calculations were performed using the ‘Zonal Statistics’ tool of ESRI’s ArcGIS 10.4 software package^50^ embedded in customized batch scripts. The zonal statistics tool produces spatial summary statistics, including mean, majority, sum, maximum, and minimum, by performing calculations on cells from a value grid (i.e., the preprocessed hydro-environmental attribute grid) within the unique spatial units of a zone grid. These zones are defined by cells with the same value, i.e., the unique lake identifier codes (IDs).

**Fig. 3:**
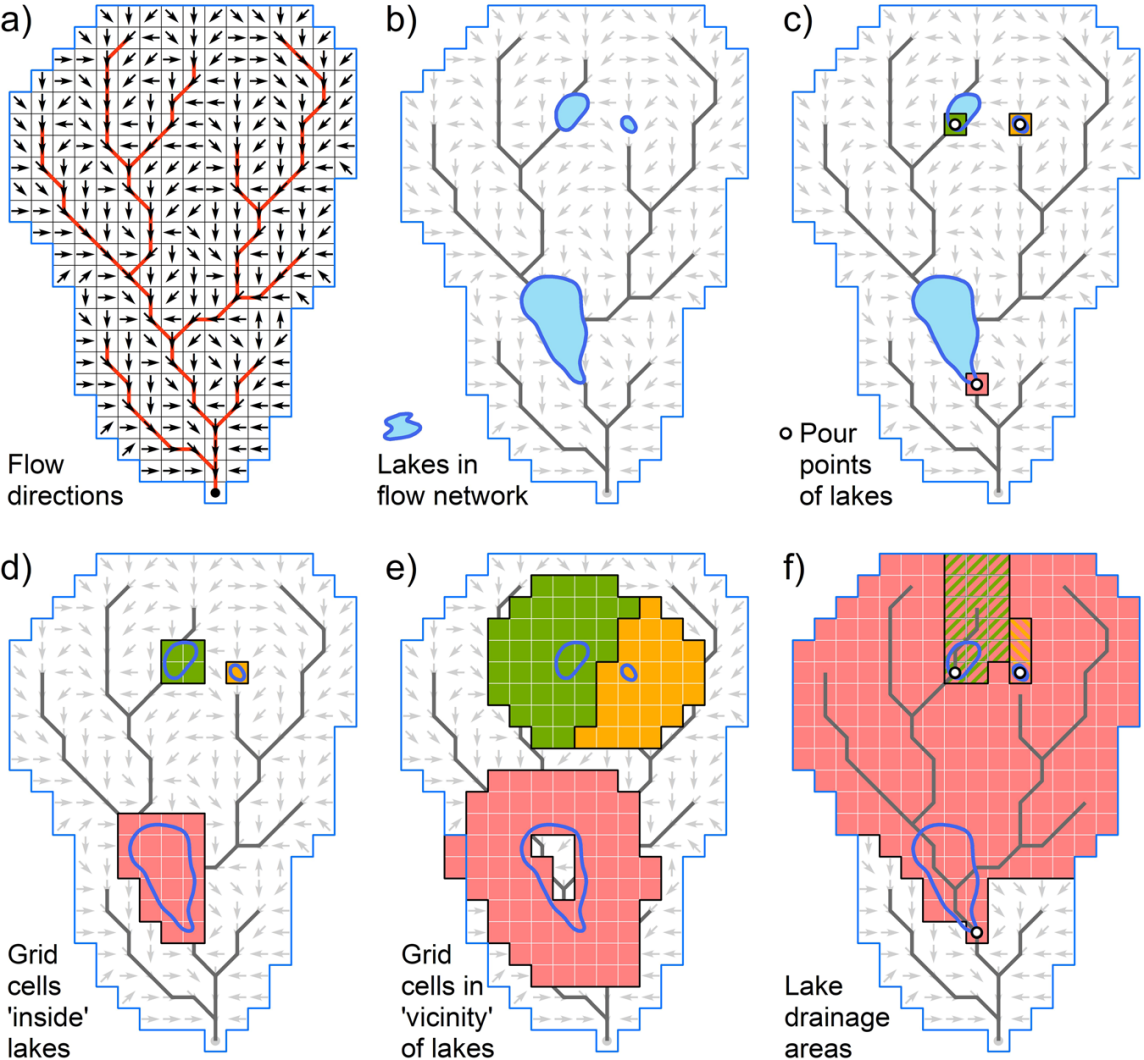
Different spatial aggregation units used for the extraction of lake attributes in LakeATLAS, and their relationship with the flow network. Panel (**a**) shows the flow directions of every pixel from which the river network (red lines) and drainage areas are derived. Panel (**b**) shows three lakes within the flow network. Other panels show the spatial extraction or aggregation zones of: (**c**) grid cells that represent lake pour points; (**d**) grid cells that represent areas ‘inside’ lakes (i.e., across the lake surface polygon); (**e**) grid cells that represent the ‘vicinity’ around lakes (i.e., in a buffer zone); and (**f**) upstream drainage areas associated with the lakes. Note that in panel (**f**) the drainage area of the lower lake also encompasses the two nested drainage areas of the upper lakes.

In preparation for the zonal statistics calculations, three zone grids of lake IDs were produced: (i) a grid showing each lake’s ID only in the grid cell that represents the lake’s pour point; (ii) a grid showing each lake’s ID in all grid cells that represent the lake’s surface polygon; and (iii) a grid showing each lake’s ID in all grid cells within a 3-km vicinity buffer around the lake (Fig. 3). The first two zone grids (i and ii) were produced because two possible zoning options were applied to derive aggregated attribute statistics within lakes depending on the nature of the attribute variable: one zoning option represents lake conditions at the pour point and the other represents lake conditions across the lake’s entire surface area. This distinction was made because some attributes are well suited to be calculated as the average or sum across the entire extent of the lake, such as precipitation or air temperature, whereas for other attributes, such as discharge, using the entire lake polygon as the zone does not deliver a meaningful metric. In this case, a single, clearly defined grid cell at the outlet of the lake creates a better representation of the lake’s overall condition (i.e., by capturing the discharge at the lake’s outlet).

For the vicinity calculations (iii), a 3-km buffer (~6 grid cells buffer distance) was chosen as a compromise between technical and functional considerations: a smaller buffer would approach the limits of the inherent mapping accuracy of some of the underpinning source data, whereas a larger buffer was deemed increasingly disassociated from the lake proper and thus less representative of its immediate surrounding characteristics. For example, in regions of high lake density in Canada, the distance between neighboring lakes is often less than 5 km and thus a 3-km buffer around one lake already includes areas that are closer to — and potentially more associated with — the neighboring lake. Furthermore, no single distance is optimal to relate characteristics surrounding lakes to *in situ* patterns and processes, and the appropriate buffer dimension may vary by lake size, type, and region as well as by the processes under study^51^. For example, Soranno *et al*.^51^ found moderately strong relationships between lake nutrients and land use within 1-km and 1.5-km buffers around lakes for 346 northern temperate lakes, ranging in size between 0.2 km^2^ and 75 km^2^, across the state of Michigan, USA. For a larger lake, i.e., Lake Taihu in China (2,250 km²), water quality in a selected bay mainly reflected land use within 2-km and 4-km buffers compared to wider areas of influence, but the land use factors that impacted water quality differed within the two buffer zones^52^. Our chosen 3-km buffer therefore also serves as a compromise to accommodate the wide range of lake sizes in LakeATLAS. Future iterations of LakeATLAS may include additional buffer sizes.

All zone grids were created at the 15 arc-second resolution to ensure proper alignment with the preprocessed attribute grids. Special attention was needed during the conversion of lakes from vector format into zonal grids to accommodate cases where multiple lake pour points or parts of multiple lake polygons occupied the same grid cell; or where a lake only occupied a partial cell area (including small lakes that do not cover even a single cell). To account for these issues and ensure that every lake was represented on each of the three zone grids, the following processing and prioritization rules were applied (for uncertainties related to these steps, see the *Technical Validation* section):

i) *‘Pour point’ zone grid*. If multiple lake pour points coincided in a single 15 arc-second grid cell (n = 7,567 affected lakes, representing 0.5% of all records), the respective pour point cell received the ID of only one representative lake. All coinciding lakes were then redefined to be associated with the same lake ID as the shared pour point cell. This ensures that the same attribute value will later be extracted to all coinciding lakes in the zonal statistics procedure. Note that in each of these cases, the ID of the smallest coinciding lake (based on its total polygon area) was assigned to the pour point cell in order to prioritize the preservation of small lakes in the zone grids of the following steps.
ii) *‘Inside’ zone grid*. First, all pour point cells and their assigned lake IDs were kept as defined in the previous step. Additional lake cells were then defined by converting the lake polygons to grid format. To increase precision, the initial conversion was conducted at a spatial resolution of 3 arc-seconds (i.e., five times finer than the target resolution of 15 arc-seconds) by creating a lake ID for any 3 arc-second pixel that was at least half (50%) covered by a single lake’s polygon. The 25 sub-pixels forming a 15 arc-second grid cell were then aggregated by assigning the majority ID to the 15 arc-second cell. In cases of ties in the majority, the ID of the smallest lake in the grid cell (based on its total polygon area) was used in order to increase the likelihood that small lakes are preserved in the grid format (whereas larger lakes are more likely to be represented by alternative grid cells). For lakes that were not uniquely preserved in this process due to exceptional geometrical constellations (n = 4 lakes; e.g., where two small lake polygons occupied the same 15 arc-second cell), they were redefined to be represented by the lake ID at their pour point location, ensuring that in the resulting zone grid every lake was at least represented by one grid cell.
  It should be noted that this conversion process, by design, defines every 15 arc-second grid cell to be a ‘lake cell’ if at least 2% of its area overlap with a lake surface polygon (i.e., 50% of at least one of its 25 sub-pixels). Grid cells that represent the shoreline of a lake are therefore assigned to be part of the lake and are included in the ‘inside’ zone calculations. This choice of slightly overrepresenting the lake surface area was made intentionally to accommodate for minor spatial uncertainties, and because a larger number of cells reduces the influence of outliers in the derivation of summary statistics. However, this expanded interpretation of a lake’s shoreline should not be confused with the specific definitions of the functional pelagic or littoral zones of a lake. In particular, we consider the lake’s polygon boundary to designate the actual land shoreline rather than the end of the pelagic zone.
iii) *‘Vicinity’ zone grid*. To define the vicinity of lakes, first a hull polygon was generated by extending each lake’s surface polygon with a ‘geodesic’ buffer going three kilometres beyond its shoreline. The resulting hull polygons were transformed into a 15 arc-second grid by converting all grid cells whose center fell within any lake hull polygon. Next, every 15 arc-second grid cell that was covered entirely by (25) lake sub-pixels at 3 arc-second resolution (see step ii) was removed from the hull grid, ensuring that the resulting buffer grid represented only the land surrounding lakes, including partial shoreline and pour point cells but excluding the actual lake surfaces. Finally, each cell within the remaining buffer grid was assigned the ID of the lake it is nearest to using the result of step (ii) as source grid for the proximity calculations. Associating each cell with a single (nearest) lake, rather than with all lakes within a distance of 3 km, prevents double-counting in global statistics (e.g., when summarizing the total population in all lake buffers for a country). However, some statistics, such as the dominant surrounding land cover, may be affected by the proximity-based partitioning of overlapping lake buffers, so users should exercise caution in the interpretation of results. Like before, lakes that were not preserved in this process due to exceptional geometrical constellations (n = 4) were represented by the lake ID at their pour point location, ensuring that in the resulting zone grid every lake was at least represented by one cell.

It should be noted that the ‘geodesic’ algorithm used to produce the buffers for LakeATLAS accounted for the Earth’s actual geoid shape by computing distances away from lakes on a curved surface, rather than using straight-line (Euclidean) distances on a flat surface. This approach was required to correct for the latitudinal distortion in the size and shape of pixels in the GCS_WGS_1984 geographic coordinate system (the coordinate system of the preprocessed grids).

Once computed, the statistics derived for the lake’s ‘pour point’, for ‘inside’ the lake (i.e., across the lake’s surface polygon) and/or within its ‘vicinity’ (i.e., within the lake’s 3-km buffer zone) were appended to all lake polygons via each lake’s unique ID (or its redefined ID in exceptional cases as outlined above). The specific spatial zones and statistics (e.g., mean, majority, sum) that were applied to extract each individual attribute are reported in the Technical Documentation of LakeATLAS.

It is important to note that the suitability or meaningfulness of a variable and its zone may differ between lake polygons and their buffers, as well as between lakes. Therefore, the interpretation and use of the provided information remain a user’s choice. For instance, the majority of the area within the 3-km buffer around a small headwater lake may extend beyond its upstream drainage area and may thus reflect the pressures exerted on the lake less closely than the 3-km buffer of a larger downstream lake which can be a more representative descriptor of the immediate zone of influence and thus the lake’s condition.

### Calculation of ‘upstream’ statistics

Statistics within lakes and in their vicinity allow for a characterization of ‘local’ hydro-environmental conditions, such as the population living in proximity to a lake shoreline or the mean annual temperature across the surface of a lake. Due to the hydrologic connectivity of the river network, however, many characteristics are better suited to an upstream perspective where the entire contributing drainage area is taken into account. For example, if an application wanted to model the water quality of a lake, this would depend both on the conditions along its shorelines (e.g., urban cover) and on conditions that originate in the entire contributing drainage area that is connected to this lake, i.e., the lake’s watershed. The latter conditions can be described with upstream statistics, such as the average population density or the total glacier, snow, or forest extent across the entire upstream watershed area that contributes to the lake. In fact, it is the very nature of freshwater systems that they depend both on local conditions and on the conditions of the entire upstream drainage area which can include parts that are far away.

To allow for the duality of both local and upstream perspectives, LakeATLAS offers pre-calculated upstream statistics for many of its characteristics. Upstream perspectives are not provided for attributes where these calculations are not meaningful, such as for ‘minimum elevation’ (as the local elevation of a lake’s water surface is identical to the lowest elevation of the entire upstream watershed), or for local attributes with an already inherent upstream perspective, such as river discharge. All upstream watershed statistics in LakeATLAS were extracted at the pour point location of each lake.

Upstream values were calculated with the standard ‘Flow Accumulation’ tool of ESRI’s ArcGIS 10.4 software package^50^ to accumulate all upstream pixel values of an attribute grid along the drainage direction map of HydroSHEDS. In order to produce upstream averages, a correction was performed to account for the latitudinal distortion in pixel sizes due to the applied geographic projection: each pixel value was first multiplied by its individual pixel area and the accumulated sum of multiplied values was then divided by the accumulated sum of pixel areas to derive an area weighted average for the watershed. In a similar way, the upstream extent of an attribute (in percent coverage), such as percent forest cover, was calculated by dividing the total area of the attribute in the upstream watershed by the total watershed area, using latitude-corrected pixel areas.

## Data Records

All hydro-environmental attributes available in version 1.0 of the LakeATLAS dataset, as well as their sources, are listed in Table 2. Most attributes with a time component (i.e., based on time series data) are provided as long-term annual averages in the attribute table of LakeATLAS, while some also include a monthly climatology (i.e., long-term monthly averages).

Each attribute offered in LakeATLAS is identified by a unique 10-character column name. This name is composed of a 3-digit key describing the name of the attribute, a 2-digit key describing the unit of the attribute, a 1-digit spatial aggregation key, and a 2-digit dimension key (plus two underscores). For example, the variable *tmp_dc_lyr* depicts air temperature (*tmp*) in degrees Celsius (*dc*) inside the lake polygon (*l*) as an annual average (*yr*), whereas the variable *aet_mm_v*06 depicts actual evapotranspiration (*aet*) in mm (*mm*) within a 3-km vicinity around the lake (*v*) as an average for the month of June (*06*), and the variable *glc_pc_u*16 depicts the global land cover extent (*glc*) in percent spatial coverage (*pc*) within the entire watershed upstream of the lake pour point (*u*) for class 16 (*16*) which represents ‘cultivated and managed areas’. All attributes refer to spatial averages across their respective aggregation unit unless explicitly stated otherwise.

Full explanations and details on the syntax of the column names, the used abbreviations, and other specifications pertaining to each attribute and its associated data source are provided in the Technical Documentation that is part of the LakeATLAS dataset^27^ (also available at https://www.hydrosheds.org/hydroatlas). In particular, the Technical Documentation includes a browsable catalog that provides an information sheet and overview map for every available variable.

## Data format and distribution 

All derived hydro-environmental attributes are provided in attribute tables associated with either the lake polygon layer or the pour point layer of HydroLAKES. LakeATLAS data are publicly available for download at https://www.hydrosheds.org/hydroatlas and as a static copy at the *figshare* data repository^27^. The LakeATLAS data layers are offered in ESRI Geodatabase and Shapefile formats. The data are projected using a geographic coordinate system with the horizontal datum of the World Geodetic System 1984 (GCS_WGS_1984). The attribute table can also be accessed as a stand-alone file in dBASE format which is included in the Shapefile format. All data are distributed with an accompanying Technical Documentation.

## Technical Validation

Like the BasinATLAS and RiverATLAS datasets (Fig. 1), the data compendium of LakeATLAS does not create new data from scratch but rather re-formats existing source data. Unless specified otherwise, all source data are used “as is”, i.e., without modification except for preprocessing (described in the *Methods* section and by Linke *et al*.^28^) and the accumulation of values along the drainage direction map of HydroSHEDS. Validation of the quality of original datasets remains with the source publications or documentations as cited in LakeATLAS.

### HydroLAKES

The reliability of LakeATLAS is largely driven by the quality and limitations of its geospatial foundation (i.e., lake polygons from HydroLAKES), which is itself dependent on its component datasets (see *Lake polygons and morphometric attributes* in *Methods* section). HydroLAKES has two main limitations with respect to mapping lake surface areas: the incomplete representation of small lakes; and its inability to represent temporal fluctuations in lake extents.

**Fig. 4:**
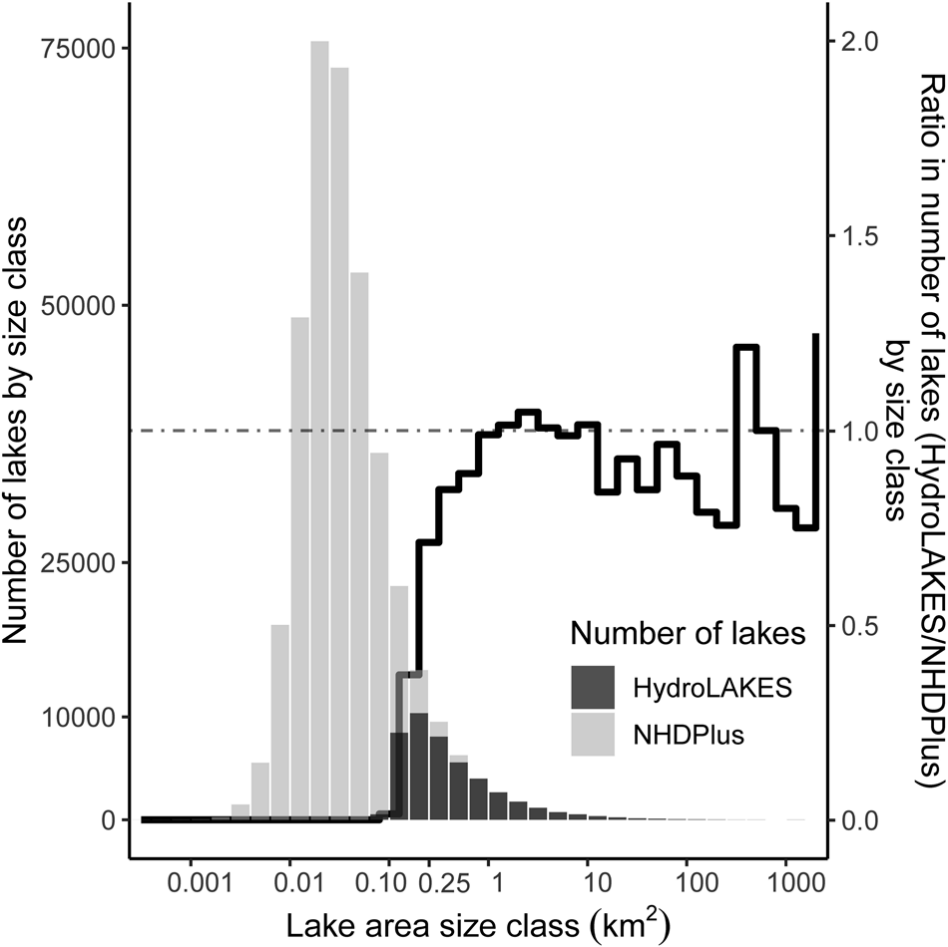
Comparison of the number of lakes represented in HydroLAKES and in the National Hydrography Dataset Plus (NHDPlus) for the contiguous United States by size class. The histogram shows the size distribution of lake polygons (left-hand axis) included in HydroLAKES (dark grey bars) compared to the NHDPlus medium resolution (light grey bars). The black line shows the ratio in the number of lake polygons for each size class between the two datasets (right-hand axis; i.e., the number of polygons in HydroLAKES divided by the number of polygons in NHDPlus, for each size class). While HydroLAKES includes lakes down to 0.10 km^2^, it comprehensively represents lake prevalence in the United States down to 0.25 km^2^ (25 ha) but misses more than half of the lakes in NHDPlus below 0.15 km^2^ (15 ha).

Given the characteristics of the underpinning source data, lakes with a length below 600 m but an area of 10 to ~25 ha (0.10 to 0.25 km^2^) are susceptible to be incompletely portrayed in HydroLAKES for most areas worldwide, except in Canada. While Canadian lake polygons were provided by the high-resolution digital Canadian hydrographic dataset CanVec^38^, most other lake polygons, in particular those below 60°N, were generated from the SWBD dataset (see *Methods*). Lake polygons from SWBD were created with orthorectified imagery of radar intensities at 1 arc-second resolution (~30 m at the equator) that was processed with semi-automated extraction protocols in combination with manual supervision and rule-based editing^14^. The technicians used land cover water masks as well as existing maps and charts from 1:50,000 to 1:1 million scales as guidance and confirmation of the presence or absence of water. In this dataset, a discontinuity affects the representation of lakes below 25 ha because the minimum size threshold used by technicians for digitizing a waterbody was a length of 600 m (and a width of 90 m)^14^. The largest lake missing due to this constraint is theoretically a round lake of 570 m in diameter spanning ~25 ha, and the proportion of omitted lakes increases with decreasing lake area. This discontinuity is apparent when comparing the size distribution of lake polygons from HydroLAKES to polygons from the reference dataset in the contiguous United States, the U.S. National Hydrography Dataset Plus (NHDPlus) medium resolution^53^ (version 2.1; scale 1:100,000; Fig. 4). In Canada, no discontinuity in lake abundance exists below 25 ha because the Canadian reference dataset (CanVec) portrays lakes down to 0.01 km^2^, an order of magnitude smaller than the surface area cut-off of 10 ha set for HydroLAKES. In Siberia, HydroLAKES polygons above 60°N latitude, i.e., in areas where SWBD is not available, were generated from the MODerate resolution Imaging Spectro-radiometer (MODIS) MOD44W Collection 5 water mask, which provides a global coverage of waterbodies at 250-m resolution^54^. As explained by Carroll *et al*.^55^, it is likely that a considerable proportion of surface waterbodies between 10 and 25 ha (≤4 pixels) were not detected in this region due to the coarse pixel size of the MODIS instrument (each pixel is ~6.25 ha in area) and were thus also missed in HydroLAKES.

The second limitation of HydroLAKES resides in its lack of temporal resolution. In contrast to more recent remote sensing products depicting monthly surface water cover, like the European Joint Research Centre (JRC) Global Surface Water Dataset^15^, a large proportion of HydroLAKES polygons are a snapshot of lake size at a given time. For instance, the lake shorelines in SWBD (i.e., below 60°N outside Canada) were delineated as they appeared at the time of the data collection in February 2000^14^; lake polygons in Europe above 60°N from the European Catchments and RIvers Network System (ECRINS) represent lake sizes in 2006 as depicted by the Corine Land Cover data (CLC2006)^56^. Therefore, some lakes may have shrunk, disappeared, grown, or appeared (including newly built reservoirs) since the acquisition of the source data depicting lake polygons in HydroLAKES. The temporal discrepancies in lake surface area may vary seasonally or sporadically, and may not be consistent across regions, such that users should use caution for areas that undergo large variations in surface water cover or where many reservoirs have been built.

### HydroSHEDS

The quality and limitations of the underpinning hydrographic framework of drainage directions is discussed in the Technical Documentation of HydroSHEDS and related products (see https://www.hydrosheds.org). The choice of various specifications, such as the pixel resolution of 15 arc-seconds, is in alignment with previous global applications of the HydroSHEDS product^45,57^ to ensure compatibility of LakeATLAS with the rest of HydroATLAS and other existing studies, data, and results. The general aim of these choices is to provide data at high spatial resolution, yet without exceeding the limits of accuracy and reliability of the underpinning global datasets, while ensuring that users can process the global data without exceptional computing facilities.

### Discharge estimates from global hydrological model

For many hydrological applications, the runoff and discharge estimates provided as part of the LakeATLAS dataset will be particularly important. Like all other attribute data in HydroATLAS, this information was provided by an existing source. Yet given its importance, we conducted a baseline evaluation of the discharge data. The long-term (1971–2000) mean discharge values provided in LakeATLAS were derived through geospatial downscaling^45^ of the 0.5-degree resolution runoff and discharge estimates of the global WaterGAP model^41^ (version 2.2 as of 2014). These estimates represent naturalized flow conditions without anthropogenic water use in the form of abstractions or impoundments^41^. After downscaling, the global total river flow into all oceans matched the original flow as modeled in WaterGAP within an error margin of 0.13%, indicating no significant distortion of large-scale totals due to the downscaling process. In addition, a validation of the downscaled discharge estimates against observations at 3,003 gauging stations globally^58^, representing river sizes from 0.004 to 180,000 m^3^ s^−1^, confirmed good overall correlations for long-term average discharges (R^2^ = 0.99 with 0.2% positive bias and a symmetric mean absolute percentage error SMAPE of 35%, improving to 13% for rivers ≥100 m^3^ s^−1^).

**Fig. 5:**
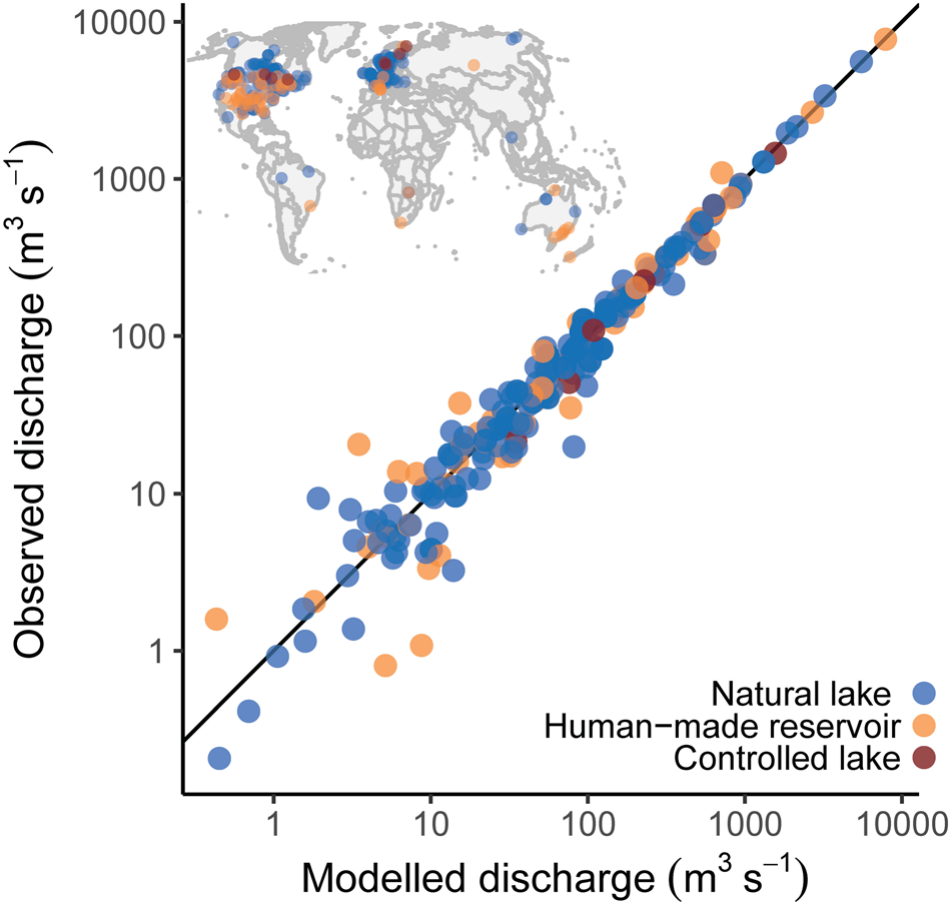
Comparison of modeled mean annual discharge in LakeATLAS to observed mean annual discharge at streamflow gauging stations monitoring lake outlets (n = 244). The inset map shows the location of the streamflow gauging stations that were included in the comparison.

We also compared the modeled discharge estimates against observed discharge for a subset of 244 stations located at or near the outlet of a lake or reservoir (Fig. 5; Table 4). Gauging stations were linked to a lake if the lake’s upstream drainage area covered at least 95% of the drainage area of the gauging station. This selection resulted in a subset of stations that monitored both natural and artificial lakes, mostly clustered in North America and Europe. The sub-comparison confirmed that discharge estimates at lake outlets were about as accurate as those of other gauging stations (R^2^ = 0.95, SMAPE = 24%), with greater accuracy for larger lakes.

**Table 4:** Summary performance statistics of LakeATLAS discharge estimates.

Lake area size class (km^2^)	R^2^	MAE* (m^3^ s^−1^)	SMAPE^$^ (%)	n
0.1–1	—	1	71	4
1–10	0.13	3	49	36
10–100	0.70	11	26	111
>100	>0.99	37	10	93
All	0.95	19	24	244

Naturalized discharge estimates from LakeATLAS (downscaled from the global WaterGAP hydrological model^41^, version 2.2) were compared to recorded discharge at 244 streamflow gauging stations near lake outlets with at least 20 years of daily records (excluding years with more than 20 missing days) for the 1971–2000 climate normal. The chosen time period matches that of the WaterGAP discharge estimates.

*Mean Absolute Error (MAE) = arithmetic mean of (observed – predicted).

^$^Symmetric Mean Absolute Percentage Error (SMAPE) = arithmetic mean of 2*(observed – predicted)/(observed + predicted).

### Preprocessing and aggregation of source data for LakeATLAS

To limit distortions and avoid the introduction of bias, the disaggregation and aggregation steps applied for the generation of LakeATLAS (see *Preprocessing of attribute data* in *Methods* section) refrain, as much as possible, from spatial interpolation methods. If original data needed to be re-projected or the resolution of original attribute datasets needed to be adjusted, the ‘nearest neighbor’ approach was applied to avoid modification of original values. This approach does not change any of the values of cells from the input raster (i.e., no averaging or median filtering is performed); it determines the location of the closest cell center on the input raster to the center of the cell in the output raster and assigns the value of that input cell to the cell on the output raster. Regional statistics and totals of the original data are thus preserved in LakeATLAS (e.g., within a lake’s drainage area).

To quantify uncertainties caused by the polygon to raster conversion of lakes, we tested the extent to which the representation of lake polygons as 15 arc-second grid cells introduced spatial inaccuracies due to misrepresentation of small (sub-pixel) lakes, overlap of multiple lakes in one pixel, or discrepancies of inside/outside associations along shorelines. For this purpose, we compared the lake surface area as calculated from the polygons against the lake surface area as calculated from the grid that defines ‘inside’ lake allocations (using latitude-corrected pixel sizes). Results showed that for lakes ≥1000 km^2^ in polygon area (n = 178), the mean overestimate was 10.8% — i.e., the sum of grid cell areas was larger than the polygon area. For lakes in the range 100–1000 km^2^ (n = 1,530), grid cell area exceeded polygon area on average by 24.1%; for lakes in the range 10–100 km^2^ (n = 14,981) by 44.0%; and for lakes in the range 1–10 km^2^ (n = 168,492) by 85.8%, which for a lake of 1 km^2^ in size corresponds to ~4 additional grid cells. The percent mismatch keeps rising for even smaller lakes, with lakes 10 ha in size showing a default overestimation of ~150% even if represented by a single 15 arc-second grid cell (~25 ha). Despite these notable mismatches, all overestimations of lake surface extent occur along the shoreline of lakes; i.e., the overestimations are spatially constrained to the distance of a single 15 arc-second grid cell and thus fall within the presumed spatial uncertainties inherent in most attribute source grids. We therefore consider these discrepancies (i.e., overestimations) to be reasonable, in particular given the intended goal of reducing the potential noise of singular pixel outliers for small lakes, which form the majority of LakeATLAS. Finally, a small number of HydroLAKES polygons fall entirely outside the land mask of HydroSHEDS (n = 793 lakes, i.e., <0.1% of all records, mostly representing lagoons or parts of estuary systems) and were thus assigned noData results for all attributes.

The quality of spatial statistics, like average air temperature within a lake or the dominant land cover class within a lake buffer, can be affected by arbitrary artefacts or outliers in the underpinning attribute data, causing uncertainties. Overall, uncertainties are largest for very small lakes that are represented by only one or few attribute pixels. Also, some attribute grids, such as soils or land cover, may inherently be affected by the presence of lakes. If lakes that are represented in a source attribute grid are not spatially congruent with the polygon for that lake in HydroLAKES (e.g., showing the same lake yet with a slight spatial offset), the determination of the true land cover or soil characteristics found in the buffer zone around the lake polygon may be affected by the ‘false’ presence of a lake water surface. Finally, the use of ‘majority’ assignments can introduce statistical bias when the results get aggregated at different scales due to an issue known as ‘modifiable areal unit problem’ (MAUP)^59^. A particularly relevant case of the problematic and scale-dependent interpretation of ‘majority’ attributes is presented in the association of each lake to a country. For countries with boundaries that are not crossed by lakes, such as Australia or any island nation, the country association of each lake is straightforward. In contrast, lakes at land borders can extend over multiple countries. For example, the Caspian Sea straddles the borders of five countries, yet the country it was assigned to is Kazakhstan as it is the country with which it overlaps the most. These types of problems need careful consideration by the user before aggregating or interpreting lake statistics for larger spatial units or different scales.

### Comparison between LakeATLAS, LakeCAT and GLCP

**Fig. 6:**
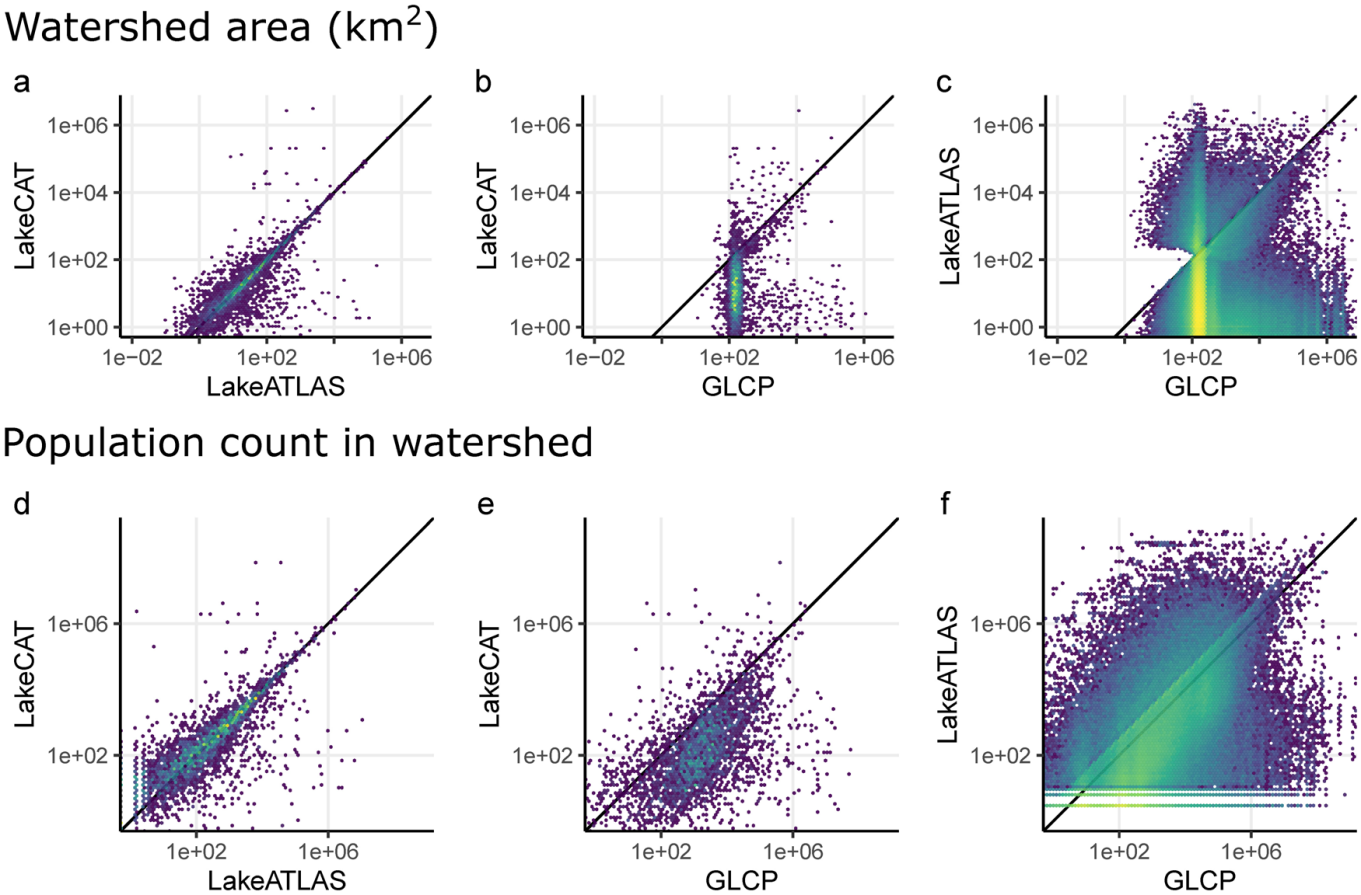
Comparison of estimates of lake watershed area and human population count in lake watersheds (or ‘lake basins’) between LakeATLAS, LakeCAT and GLCP. Only lake polygons that overlap for at least 90% of their respective surface areas across datasets were included in the comparisons with LakeCAT (**a,b**, **d,e**; n = 3,975). Because LakeATLAS and GLCP both rely on lake polygons from HydroLAKES, we compared watershed area and population attributes for all lakes between these two datasets (**c**, **f**; n = 1,422,499). All axes are logarithm-transformed and black lines represent the identity line (1:1 line of equality) for each plot.

Given the importance of a lake’s upstream drainage area on its water quality and functioning, it is crucial to correctly delineate lake watersheds. For LakeATLAS, we used network routing algorithms to determine the hydro-environmental characteristics of the entire area upstream of each lake’s pour point based on the drainage direction grids of HydroSHEDS (see *Calculation of ‘upstream’ statistics* in *Methods* section). A similar approach was applied in LakeCAT to compute the characteristics of lake watersheds for the contiguous United States, albeit using drainage direction grids at a much higher resolution (1 arc-second for LakeCAT^21^ vs. 15 arc-seconds for LakeATLAS). To compare LakeATLAS with LakeCAT, we spatially joined lake polygons from both datasets, matching lakes that overlapped for at least 90% of their respective area. Based on this subset of lakes, we found that watershed areas delineated for LakeATLAS using lake pour points and HydroSHEDS drainage direction grids corresponded relatively well to watershed areas in LakeCAT (Fig. 6a; log-log least-square regression R^2^ = 0.61, SMAPE = 51%, n = 3,975). The largest discrepancies were typically caused by different interpretations of lake pour points that were located near confluences between streams of substantially different size categories. In these situations, even a minor spatial mismatch can assign a lake to the small drainage basin of the tributary stream instead of the large basin of the mainstem river, or vice versa. Small lakes were more frequently affected by this issue.

Like LakeATLAS, the GLCP database^24^ relies on the lake polygons from HydroLAKES. In contrast to LakeATLAS and LakeCAT, however, GLCP assigns climate and population statistics to ‘lake basins’ that were not strictly computed from the lakes’ actual hydrologic watersheds (i.e., the upstream areas that drain into each lake). Instead, GLCP uses a spatial proxy for each lake that is defined by the smallest sub-basin that encloses the lake in its entirety^24^, based on the sub-basin geometry provided in the HydroBASINS dataset^45^. As HydroBASINS subdivides watersheds based on stream confluences rather than lake outlets, the spatial representativeness of the resulting ‘lake basins’ remains ambiguous. On the one hand, substantial parts of the associated basin may be located downstream of the lake; this issue is particularly important for headwater lakes with small drainage areas (note that even for the smallest lake size class, the median ‘lake basin’ size in GLCP is 156 km^2^). On the other hand, for lakes with large watersheds, the basins used in GLCP may not comprise the entire upstream drainage area but only part of it. Therefore, the physical meaning of the ‘lake basin’ associated with each lake in GLCP is inconsistent across lake sizes and landscape configurations, and neither represents the full hydrologic drainage area nor a clearly defined area-of-influence within a given buffer distance around the lake. This potential mismatch is illustrated by comparing the area of ‘lake basins’ identified in GLCP to the hydrologic drainage areas of lakes determined from LakeCAT and LakeATLAS. Differences between GLCP and LakeCAT (Fig. 6b**;** log-log least-square regression R^2^ = 0.04, SMAPE = 146%, n = 3,970) result from both differences in lake polygons and basin association methods. Differences between GLCP and LakeATLAS (Fig. 6c; log-log least-square regression R^2^ = 0.02, SMAPE = 171%, n = 1,422,499; see ref. ^24^ for details on the 5,189 HydroLAKES polygons excluded from GLCP) only stem from differences in basin association methods as these two datasets both rely on HydroLAKES surface polygons. For both comparisons (Fig. 6b,c), GLCP ‘lake basins’ tend to overstate actual lake drainage areas for lakes with watersheds smaller than ~100 km^2^, which reflects about the smallest average size of sub-basin units available in HydroBASINS. In contrast, GLCP tends to understate actual lake drainage areas for larger watersheds, as GLCP only picks the smallest sub-basin unit that entirely contains the lake rather than the entire drainage area.

The differences in watershed delineation (or association) between the three datasets also have implications for the computation of watershed-level statistics. This is for example the case for estimates of human population counts within the lake watershed (or ‘lake basin’) in 2010, an attribute that all three datasets share, although LakeCAT contains statistics from a different source of population data than LakeATLAS and GLCP. LakeCAT relies on the United States decennial census^60^ while LakeATLAS and GLCP rely on the Gridded Population of the World (GPW) dataset^61^, version 4. Despite relying on a different data source, lake polygons, and resolution, LakeATLAS provides a relatively accurate estimate (as compared to LakeCAT) of the population count within lake watersheds (Fig. 6d; log-log least square regression R^2^ = 0.66, SMAPE = 92%, n = 3,975). GLCP, within its ‘lake basin’, overstates the population count within the actual hydrologic drainage area (i.e., upstream) compared to LakeCAT (Fig. 6e) for 85% of lakes, particularly for lakes with smaller watersheds (log-log least square regression R^2^ = 0.35, SMAPE = 148%, n = 3,970). Similarly, despite relying on the same source of data as LakeATLAS, GLCP estimates of population counts in ‘lake basins’ are not comparable to LakeATLAS estimates in the lakes’ actual drainage areas (Fig. 6f).

## Usage Notes

LakeATLAS offers a large variety of hydro-environmental attributes intended for a broad range of user applications. It remains the user’s responsibility to decide whether certain attributes, statistics, or scales are meaningful and appropriate. For example, the association of a large lake to a single country based on spatial majority may be adequate for a lake that is entirely or mostly within the country but can be highly misleading for a transboundary lake spanning multiple countries. Similarly, the association of coarser scale attributes, such as national GDP values, to small lakes may be meaningful for statistical assessment purposes, yet it will not realistically represent small-scale spatial patterns. More generally, users are expected to inform themselves on the meaning, quality, and relevant uses of the source data by consulting the primary literature associated with each attribute.

Beyond the existing attribute columns contained in LakeATLAS, users can extract a variety of inherent information by applying their own post-processing algorithms and cross-calculations. For example, attributes can be analyzed by comparing results across different scales, such as comparing lakes with relatively unpopulated shorelines but high population densities in their watershed to lakes with densely populated shorelines but a sparsely populated watershed — identifying the importance of local versus distal factors. Similarly, attributes can be summarized by other attributes, such as the percentage of lakes within protected areas per country or per freshwater ecoregion. Finally, attributes can also be normalized using the existing information of multiple columns. For example, discharge can be divided by upstream watershed area in order to calculate ‘specific discharge (per km^2^)’; or by upstream population numbers in order to calculate ‘water availability per person’ in the lake’s drainage area.

The BasinATLAS and RiverATLAS vector datasets are both derived from the same underpinning hydrography of HydroSHEDS that was used for the identification of lake pour points. Therefore, all three components of HydroATLAS (BasinATLAS, RiverATLAS, and LakeATLAS) are mutually linkable via uniquely defined spatial relationships. For example, every river reach is associated with one or more lakes (one-to-many relationship), every lake’s pour point is associated with a single river reach (one-to-one relationship), and every river reach or lake falls within a sub-basin (many-to-one relationship). Through these relationships, a lake can be associated with the river network characteristics of the river reach at its pour point, such as the distance from the upstream headwater source or from the final downstream pour point (i.e., at the ocean or at the most downstream pixel of an endorheic basin). Similarly, LakeATLAS is generally compatible with the growing list of other raster and vector datasets that are built from, or linked to, the hydrographic framework of HydroSHEDS. Examples of such datasets encompass a global assessment of the free-flowing status of rivers, including an estimate of their sediment transport^62^, and a range of aquatic species compilations, including continental maps produced by IUCN^63,64^. Other datasets that rely on HydroLAKES as a geospatial foundation, including GLCP, can also be linked to LakeATLAS via lake polygon IDs.

Intensive efforts have been made to verify the licenses of the underpinning source datasets, and specific permissions were obtained from individual authors if needed, in order to release all derived attribute columns of LakeATLAS (version 1.0) under either a Creative Commons Attribution 4.0 International License (CC-BY 4.0) or an Open Data Commons Open Database License (ODbL 1.0), both permitting reuse of the data for any purpose including commercial. LakeATLAS users are requested to honor the individual reference suggestions of the source data providers; hence citations and acknowledgements should be made to both the original data source(s) and the LakeATLAS compendium. For example, the following template illustrates a reference to precipitation data sourced from LakeATLAS: “Precipitation data from the WorldClim v1.4 database (Hijmans *et al*. 2005) has been used in the spatial format of LakeATLAS v1.0 (Lehner *et al*. 2022).” Detailed information regarding the license and reference(s) for each attribute column is provided in the Technical Documentation of LakeATLAS and in Table 2.

## Data Availability

All data processing steps were performed using native tools and/or customized batch processing within ESRI’s ArcGIS 10.4 software package^50^ in a dedicated computing setup (64-bit processing). The two core tools applied were ‘Zonal Statistics’ and ‘Flow Accumulation’. To support repetitive tasks of this work, a multitude of adjusted batch routines were developed as needed, mostly defining input and output path names for the standard tools and to handle internal object IDs. No stand-alone programming code was created that allows automatic processing of new data into the format of LakeATLAS. This is in alignment with the premise of our work, i.e., to produce standardized data by applying tedious, individual, and customized GIS steps specific to every input dataset so that other users do not have to repeat these time-consuming manual iterations.

## References

[1] Shiklomanov, I. A. & Rodda, J. C. *World water resources at the beginning of the twenty-first century*. (Cambridge University Press, 2003).

[2] Biggs J, von Fumetti S, Kelly-Quinn M (2017). The importance of small waterbodies for biodiversity and ecosystem services: implications for policy makers. Hydrobiologia.

[3] Heino J (2021). Lakes in the era of global change: moving beyond single-lake thinking in maintaining biodiversity and ecosystem services. Biol. Rev..

[4] Janssen ABG (2021). Shifting states, shifting services: linking regime shifts to changes in ecosystem services of shallow lakes. Freshw. Biol..

[5] Knoll LB (2019). Consequences of lake and river ice loss on cultural ecosystem services. Limnol. Oceanogr. Lett..

[6] Sterner RW (2020). Ecosystem services of Earth’s largest freshwater lakes. Ecosyst. Serv..

[7] Reynaud A, Lanzanova D (2017). A global meta-analysis of the value of ecosystem services provided by lakes. Ecol. Econ..

[8] Cooley SW, Ryan JC, Smith LC (2021). Human alteration of global surface water storage variability. Nature.

[9] Downing JA (2009). Global limnology: up-scaling aquatic services and processes to planet Earth. SIL Proceedings, 1922–2010.

[10] Tranvik LJ, Cole JJ, Prairie YT (2018). The study of carbon in inland waters—from isolated ecosystems to players in the global carbon cycle. Limnol. Oceanogr. Lett..

[11] Balsamo G (2012). On the contribution of lakes in predicting near-surface temperature in a global weather forecasting model. Tellus A Dyn. Meteorol. Oceanogr..

[12] DelSontro T, Beaulieu JJ, Downing JA (2018). Greenhouse gas emissions from lakes and impoundments: upscaling in the face of global change. Limnol. Oceanogr. Lett..

[13] Beaulieu JJ (2020). Methane and carbon dioxide emissions from reservoirs: controls and upscaling. J. Geophys. Res. Biogeosciences.

[14] Slater JA (2006). The SRTM data “finishing” process and products. Photogramm. Eng. Remote Sens..

[15] Pekel J-F, Cottam A, Gorelick N, Belward AS (2016). High-resolution mapping of global surface water and its long-term changes. Nature.

[16] Verpoorter C, Kutser T, Seekell DA, Tranvik LJ (2014). A global inventory of lakes based on high-resolution satellite imagery. Geophys. Res. Lett..

[17] Pickens AH (2020). Mapping and sampling to characterize global inland water dynamics from 1999 to 2018 with full Landsat time-series. Remote Sens. Environ..

[18] Messager ML, Lehner B, Grill G, Nedeva I, Schmitt O (2016). Estimating the volume and age of water stored in global lakes using a geo-statistical approach. Nat. Commun..

[19] Tickner D (2020). Bending the curve of global freshwater biodiversity loss: an emergency recovery plan. Bioscience.

[20] Downing JA, Polasky S, Olmstead SM, Newbold SC (2021). Protecting local water quality has global benefits. Nat. Commun..

[21] Hill RA, Weber MH, Debbout RM, Leibowitz SG, Olsen AR (2018). The Lake-Catchment (LakeCat) Dataset: characterizing landscape features for lake basins within the conterminous USA. Freshw. Sci..

[22] Soranno PA (2017). LAGOS-NE: a multi-scaled geospatial and temporal database of lake ecological context and water quality for thousands of US lakes. Gigascience.

[23] Toptunova O, Choulga M, Kurzeneva E (2019). Status and progress in global lake database developments. Adv. Sci. Res..

[24] Meyer MF, Labou SG, Cramer AN, Brousil MR, Luff BT (2020). The global lake area, climate, and population dataset. Sci. Data.

[25] Kling GW, Kipphut GW, Miller MM, O’Brien WJ (2000). Integration of lakes and streams in a landscape perspective: the importance of material processing on spatial patterns and temporal coherence. Freshw. Biol..

[26] Fergus CE (2017). The freshwater landscape: lake, wetland, and stream abundance and connectivity at macroscales. Ecosphere.

[27] Lehner B, Messager ML, Korver MC, Linke S (2022). figshare.

[28] Linke S (2019). Global hydro-environmental sub-basin and river reach characteristics at high spatial resolution. Sci. data.

[29] Fergus CE (2021). National framework for ranking lakes by potential for anthropogenic hydro-alteration. Ecol. Indic..

[30] Bracht-Flyr B, Istanbulluoglu E, Fritz S (2013). A hydro-climatological lake classification model and its evaluation using global data. J. Hydrol..

[31] Soranno PA (2010). Using landscape limnology to classify freshwater ecosystems for multi-ecosystem management and conservation. Bioscience.

[32] McCullough IM, Skaff NK, Soranno PA, Cheruvelil KS (2019). No lake left behind: how well do U.S. protected areas meet lake conservation targets?. Limnol. Oceanogr. Lett..

[33] Stanley EH (2019). Biases in lake water quality sampling and implications for macroscale research. Limnol. Oceanogr..

[34] Hanson PC, Weathers KC, Kratz TK (2016). Networked lake science: how the Global Lake Ecological Observatory Network (GLEON) works to understand, predict, and communicate lake ecosystem response to global change. Inl. Waters.

[35] Lottig NR, Carpenter SR (2012). Interpolating and forecasting lake characteristics using long-term monitoring data. Limnol. Oceanogr..

[36] Filazzola A (2020). A database of chlorophyll and water chemistry in freshwater lakes. Sci. Data 2020 71.

[37] Lehner, B. & Messager, M. L. *HydroLAKES - Technical Documentation Version 1.0*. https://data.hydrosheds.org/file/technical-documentation/HydroLAKES_TechDoc_v10.pdf (2016).

[38] Natural Resources Canada. CanVec Hydrography: Waterbody Features. Version 12.0. https://ftp.maps.canada.ca/pub/nrcan_rncan/vector/canvec (2013).

[39] Lehner B, Verdin K, Jarvis A (2008). New global hydrography derived from spaceborne elevation data. Eos, Trans. AGU.

[40] Farr TG, Kobrick M (2000). Shuttle radar topography mission produces a wealth of data. Eos, Trans. AGU.

[41] Müller Schmied H (2021). The global water resources and use model WaterGAP v2.2d: model description and evaluation. Geosci. Model Dev..

[42] Beck HE (2017). Global evaluation of runoff from 10 state-of-the-art hydrological models. Hydrol. Earth Syst. Sci..

[43] Alcamo J (2003). Development and testing of the WaterGAP 2 global model of water use and availability. Hydrol. Sci. J..

[44] Döll P, Kaspar F, Lehner B (2003). A global hydrological model for deriving water availability indicators: model tuning and validation. J. Hydrol..

[45] Lehner B, Grill G (2013). Global river hydrography and network routing: baseline data and new approaches to study the world’s large river systems. Hydrol. Process..

[46] Fick SE, Hijmans RJ (2017). WorldClim 2: new 1-km spatial resolution climate surfaces for global land areas. Int. J. Climatol..

[47] Hengl T (2017). SoilGrids250m: Global gridded soil information based on machine learning. PLoS One.

[48] Zhang X (2021). GLC_FCS30: Global land-cover product with fine classification system at 30 m using time-series Landsat imagery. Earth Syst. Sci. Data.

[49] Buchhorn M (2020). Zenodo.

[50] ESRI. ArcGIS Desktop: Release 10.4.1 (Environmental Systems Research Institute, Redlands, CA, USA, 2016).

[51] Soranno PA, Cheruvelil KS, Wagner T, Webster KE, Bremigan MT (2015). Effects of land use on lake nutrients: the importance of scale, hydrologic connectivity, and region. PLoS One.

[52] Su ZH, Lin C, Ma RH, Luo JH, Liang QO (2015). Effect of land use change on lake water quality in different buffer zones. Appl. Ecol. Environ. Res..

[53] Brakebill, J. W., Schwarz, G. E. & Wieczorek, M. E. *An enhanced hydrologic stream network based on the NHDPlus medium resolution dataset*. *Scientific Investigations Report*10.3133/sir20195127 (2020).

[54] Carroll, M., Townshend, J., DiMiceli, C., Noojipady, P. & Sohlberg, R. Global raster water mask at 250 meter spatial resolution, Collection 5: MOD44W MODIS Water Mask. *College Park, Maryland: University of Maryland* (2009).

[55] Carroll ML, Townshend JR, DiMiceli CM, Noojipady P, Sohlberg RA (2009). A new global raster water mask at 250 m resolution. Int. J. Digit. Earth.

[56] European Environment Agency (EEA). European Catchments and Rivers Network System (ECRINS), https://www.eea.europa.eu/data-and-maps/data/european-catchments-and-rivers-network (2012).

[57] Ouellet Dallaire C, Lehner B, Sayre R, Thieme M (2019). A multidisciplinary framework to derive global river reach classifications at high spatial resolution. Environ. Res. Lett..

[58] Global Runoff Data Centre (GRDC). River discharge data. *Federal Institute of Hydrology, 56068 Koblenz, Germany*, https://www.bafg.de/GRDC (2014).

[59] Openshaw, S. The modifiable areal unit problem. In *Quantitative Geography: A British View* (eds. Wrigley, N. & Bennett, R.) 60–69 (Routledge and Kegan Paul, Andover, 1981).

[60] United States Census Bureau. 2010 Census. ftp://ftp2.census.gov/geo/tiger (2010).

[61] (2016).

[62] Grill G (2019). Mapping the world’s free-flowing rivers. Nature.

[63] Allen, D. J. *et al*. *The Diversity of Life in African Freshwaters: Under Water, Under Threat: an Analysis of the Status and Distribution of Freshwater Species Throughout Mainland Africa*. (IUCN, 2011).

[64] Markovic D (2014). Europe’s freshwater biodiversity under climate change: distribution shifts and conservation needs. Divers. Distrib..

[65] Fluet-Chouinard E, Lehner B, Rebelo L-M, Papa F, Hamilton SK (2015). Development of a global inundation map at high spatial resolution from topographic downscaling of coarse-scale remote sensing data. Remote Sens. Environ..

[66] Lehner B (2011). High‐resolution mapping of the world’s reservoirs and dams for sustainable river‐flow management. Front. Ecol. Environ..

[67] Fan Y, Li H, Miguez-Macho G (2013). Global patterns of groundwater table depth. Science.

[68] Robinson N, Regetz J, Guralnick RP (2014). EarthEnv-DEM90: A nearly-global, void-free, multi-scale smoothed, 90m digital elevation model from fused ASTER and SRTM data. ISPRS J. Photogramm. Remote Sens..

[69] Metzger MJ (2013). A high-resolution bioclimate map of the world: a unifying framework for global biodiversity research and monitoring. Glob. Ecol. Biogeogr..

[70] Hijmans RJ, Cameron SE, Parra JL, Jones PG, Jarvis A (2005). Very high resolution interpolated climate surfaces for global land areas. Int. J. Climatol..

[71] Zomer RJ, Trabucco A, Bossio DA, Verchot LV (2008). Climate change mitigation: a spatial analysis of global land suitability for clean development mechanism afforestation and reforestation. Agric. Ecosyst. Environ..

[72] Trabucco A, Zomer RJ, Bossio DA, van Straaten O, Verchot LV (2008). Climate change mitigation through afforestation/reforestation: a global analysis of hydrologic impacts with four case studies. Agric. Ecosyst. Environ..

[73] Trabucco, A. & Zomer, R. J. Global soil water balance geospatial database. *CGIAR Consortium for Spatial Information*, https://cgiarcsi.community/data/global-high-resolution-soil-water-balance (2010).

[74] Hall DK, Riggs GA, Salomonson V (2016). 2002–2015.

[75] Bartholomé E, Belward AS (2005). GLC2000: a new approach to global land cover mapping from Earth observation data. Int. J. Remote Sens..

[76] Ramankutty N, Foley JA (1999). Estimating historical changes in global land cover: Croplands from 1700 to 1992. Global Biogeochem. Cycles.

[77] Lehner B, Döll P (2004). Development and validation of a global database of lakes, reservoirs and wetlands. J. Hydrol..

[78] Ramankutty, N., Evan, A. T., Monfreda, C. & Foley, J. A. Farming the planet: 1. Geographic distribution of global agricultural lands in the year 2000. *Global Biogeochem. Cycles* **22**, (2008).

[79] Siebert S (2015). A global data set of the extent of irrigated land from 1900 to 2005. Hydrol. Earth Syst. Sci..

[80] (2012).

[81] Gruber S (2012). Derivation and analysis of a high-resolution estimate of global permafrost zonation. Cryosphere.

[82] UNEP-WCMC & IUCN. The World Database on Protected Areas, http://www.protectedplanet.net (2014).

[83] Dinerstein E (2017). An ecoregion-based approach to protecting half the terrestrial realm. Bioscience.

[84] Abell R (2008). Freshwater ecoregions of the world: a new map of biogeographic units for freshwater biodiversity conservation. Bioscience.

[85] Hengl T (2014). SoilGrids1km—global soil information based on automated mapping. PLoS One.

[86] Hartmann J, Moosdorf N (2012). The new global lithological map database GLiM: a representation of rock properties at the Earth surface. Geochem. Geophys. Geosyst..

[87] Williams PW, Ford DC (2006). Global distribution of carbonate rocks. Zeitschrift für Geomorphologie Suppl..

[88] Borrelli P (2017). An assessment of the global impact of 21st century land use change on soil erosion. Nat. Commun..

[89] Pesaresi, M. & Freire, S. GHS Settlement grid following the REGIO model 2014 in application to GHSL Landsat and CIESIN GPW v4-multitemporal (1975-1990-2000-2015). European Commission, Joint Research Centre (JRC), https://data.europa.eu/data/datasets/jrc-ghsl-ghs_smod_pop_globe_r2016a (2016).

[90] Doll, C. N. H. CIESIN thematic guide to night-time light remote sensing and its applications. CIESIN http://sedac.ciesin.columbia.edu/binaries/web/sedac/thematic-guides/ciesin_nl_tg.pdf (2008).

[91] Meijer JR, Huijbregts MAJ, Schotten KCGJ, Schipper AM (2018). Global patterns of current and future road infrastructure. Environ. Res. Lett..

[92] Venter O (2016). Global terrestrial Human Footprint maps for 1993 and 2009. Sci. data.

[93] University of Berkeley. Database of global administrative areas (GADM). University of Berkeley, Museum of Vertebrate Zoology and the International Rice Research Institute, http://www.gadm.org (2012).

[94] Kummu M, Taka M, Guillaume JHA (2018). Gridded global datasets for gross domestic product and Human Development Index over 1990–2015. Sci. data.

